# Decoding the Molecular Grammar of TIA1-Dependent Stress Granules in Proteostasis and Welander Distal Myopathy Under Oxidative Stress

**DOI:** 10.3390/cells13231961

**Published:** 2024-11-27

**Authors:** Isabel Alcalde-Rey, Beatriz Ramos Velasco, José Alcalde, José M. Izquierdo

**Affiliations:** Centro de Biología Molecular Severo Ochoa (CBM), Consejo Superior de Investigaciones Científicas, Universidad Autónoma de Madrid (CSIC/UAM), C/Nicolás Cabrera 1, 28049 Madrid, Spain; isabelalcalderey@gmail.com (I.A.-R.); bramos@cbm.csic.es (B.R.V.); jalcalde@cbm.csic.es (J.A.)

**Keywords:** TIA1, TIAR, stress granules, Welander distal myopathy, oxidative stress

## Abstract

T-cell intracellular antigen 1 (TIA1) is an RNA-binding protein (RBP) that plays a multifunctional role in RNA metabolism. TIA1 has three RNA-Recognition Motifs (RRMs) and a prion-like carboxyl C-terminal domain (LCD) with intrinsically disordered regions (IDR) implicated in the dynamics (i.e., formation, assembly, and disassembly) of transient RNA-protein aggregates known as stress granules (SGs). A protein related to TIA1 is its paralog TIA1-related/like protein (TIAR/TIAL1), whose amino acid sequence, structural organisation, and molecular and cellular functions are highly conserved with TIA1. Both proteins are the main components of SGs, which are non-membranous RNA-protein condensates formed under stress to promote cell survival. Welander distal myopathy (WDM) is a late-onset muscular dystrophy that has been linked to a single-nucleotide substitution (c.1362G>A; p.E384K) in the gene encoding the TIA1 protein, which impacts TIA1-dependent SGs dynamics. Herein, we have analysed cellular and molecular aspects by targeting mutagenesis to position 384 to understand its molecular grammar in an amino acid/proteinogenic-dependent or -independent manner under oxidative stress. The observations suggest differential, even opposing, behaviours between TIA1 and TIAR in the presence of specific amino acids with negative and positive charges, and also uncharged acids, at equivalent positions of TIA1 and TIAR, respectively. Collectively, these findings illustrate a characteristic molecular grammar of TIAR- and TIA1-dependent SGs under oxidative conditions, suggesting a gain of versatility between two structurally and functionally highly conserved/related proteins.

## 1. Introduction

T-cell intracellular antigen 1 (TIA1) is an RNA-binding protein (RBP) that regulates gene expression and is involved in stress granule (SG) biology [1,2,3,4,5,6,7,8,9,10,11,12]. Since its discovery, this protein has been studied for its regulatory role in the control of gene expression flux, cellular RNA dynamics and metabolism, and various cellular and biological processes that are directly or indirectly associated with human pathologies due to alterations of the normal action of this multifunctional master regulator [1,2,3,4,5,6,7,8,9,10,11,12].

The human *TIA1* gene, located on chromosomal region 2p13 [13,14], consists of 13 exons that, through alternative splicing of exon 5, generate two major isoforms [13,14,15]. TIA1 has three RNA-Recognition Motifs (RRM1-3) and a low-complexity C-terminal domain (LCD), rich in glutamine and asparagine, which favours protein–protein interactions [14,16,17]. RRM2 and RRM3 are the most conserved and are mainly responsible for RNA binding and recognition, while the LCD is more variable throughout the structural orthologs identified in all vertebrate groups [12,14,16,17].

TIA1-related/like protein (TIAR/TIAL1) is the TIA1 paralog [18], consisting of three RRM domains and an LCD. The homology/identity between the RRM domains of these two proteins is very high at 85% (79% for RRM1, 89% for RRM2, and 91% for RRM3); however, their LCDs only share 51% homology [14,16]. Regarding their distribution in mice [14] and humans [15], it was found that, while the mRNA of both proteins was widely expressed in the tissues in humans, TIAR and TIA1 were ubiquitously expressed in the nucleus and cytoplasm throughout the organism [8,18,19,20,21,22]. These proteins are partially redundant, one can substitute the absence of the other, but each has independent and exclusive aspects in several situations, such as embryonic development, processes associated with stressful situations, or pathological dysfunctions [2,8,18,19,20,21,22].

Both TIA proteins are multi-functional regulators/modulators with an important role in the flux of genetic expression [12,22]. TIA1 and TIAR participate in several cellular processes and biological programmes, like apoptosis, autophagy, cellular growth, mitochondrial dynamics, stress response, inflammation, embryogenesis, tumorigenesis, and viral response [12,22]. On the other hand, TIA1 expression, function, or regulation disorders are associated with different illnesses, among which are neurological diseases, viral infections, post-traumatic stress, cancer, and muscular diseases like Welander distal myopathy (WDM) [12,22,23,24].

Additionally, TIA1 plays a main role in the nucleation, formation, and dynamics of SGs [2,25,26]. These are non-membranous aggregates containing mRNAs, non-coding RNAs, and pre-initiation complex components of the canonical translation machinery, as well as other RBPs and cellular components related to transcription, replication, epigenetics, and cell signalling pathways [25,26,27,28]. The LCD, rich in glutamine and asparagine [26,27], is necessary for interaction with other proteins and SG assembly. Indeed, TIA1 is a canonical component of granules that are generated under stress conditions (e.g., oxidative stress, heat shock, osmotic stress, etc.) [2,25,26,27,28].

The formation, assembly, and disassembly dynamics of these SGs function through complex biophysical events known as liquid–liquid phase separation (LLPS) [28,29,30,31]. Today, the molecular details that drive LLPS leading to the formation of aggregates, the molecular basis of SGs, are not well understood. However, talk of “molecular grammar” has begun, according to which there are interactions between certain amino acids that could favour or frustrate the process [3]. Moreover, electrostatic interactions between RRM and LCDs should be considered as a main factor throughout the process, influenced by positively or negatively charged amino acids, favouring attraction or repulsion. In summary, amino acid properties (polarity, aromaticity, charge, and size) exert an essential influence on the interplay between peptide domains to trigger LLPS, which is the origin of SG assembly [32,33].

WDM is a dominantly inherited autosomal muscular dystrophy that manifests late in life (40–60 years) [34,35,36]. The disorder first manifests by affecting finger and hand muscles and, in time, disturbs the distal muscles of the lower extremities, impairing fine motor skills and movement [34,35]. The wild-type (WT) gene harbours guanine at position 1362, which is substituted by adenine in the mutant version. This change alters the codon, which originally encoded glutamic acid (E) at position 384, to code for lysine (K) in the mutated protein present in WDM (c.1362G>A; p.E384) [24,35,37].

In the WDM-related TIA1 variant, the number and area of SGs increased in cultured HeLa cells [24]. Hackman’s lab proposed a model to explain this pathology, in which the increased tendency of mutant TIA1 to aggregate could lead to the accumulation of remnants of these granules in muscle tissue, leading to the aggregation of other proteins, which may be the underlying cause of the disease. They also found that the attenuated assembly or disassembly of SGs could cause the dysregulation of protein expression in stress situations, causing proteotoxic stress and, over time, the accumulation of defective proteins that would lead cells to autophagy. Thus, the effects of the mutation were previously analysed in a heterogeneous cell model [11,36]. These observations manifested that TIA1-dependent SGs were generated faster, and they were larger and more stable, thus their disassembly was slower. Furthermore, effects on cellular signalling and autophagy, mitophagy, and apoptosis responses were seen. On the other hand, the effect of the WDM mutation on the pre-mRNA splicing of Survival Motor Neuron 2 (SMN2) was mild-to-moderate [11].

To address the molecular details governing TIA1-dependent SG formation and dynamics, new TIA1 variants were generated with each proteinogenic amino acid substitution at position 384 of the TIA1 protein isoform. This approach aimed to understand the molecular grammar of TIA1-dependent SGs. Furthermore, as WDM is exclusively associated with a mutation in *TIA1* and not with its paralog *TIAR*, we decided to investigate whether this specific mutation and other derivative mutations in *TIAR* have a phenotype in TIAR-dependent SGs. Our results suggest that this situation cannot be extrapolated to TIAR, the TIA1 paralog.

## 2. Materials and Methods

### 2.1. Cell Culture

HEK293 Flp-In T-REx cells (HEK-Flp cells; Invitrogen, Waltham, MA, USA) were cultured in *Dulbecco’s modified Eagle’s medium* (DMEM) with 2% Foetal Bovine Serum (FBS) in an incubator (Thermo Electron Corporation, Waltham, MA, USA) at 37 °C, with 95% humidity and 5% CO_2_ [6,11,38]. All cultures were regularly diluted upon reaching 90% confluence. To visualise the cells with confocal microscopy, we treated cover slides with Poly-L-Lysine solution (0.01%) (Sigma, San Luis, MO, USA) during 3–4 h for greater adherence [39].

### 2.2. TIA1 and TIAR Mutant Generation

pcDNA-5/FRT/TO-GFP-TIA1^E/WT^ and pcDNA-5/FRT/TO-GFP-TIA1^K/WDM^ plasmids, which incorporate glutamic acid and lysine, respectively, at position 384 of the TIA1 protein (the major isoform of TIA1 expressed in Flp-HEK cells), were previously generated [11]. pcDNA-5/FRT/TO-GFP-TIAR^Q/WT^ of the TIAR protein, which includes glutamine at residue 373, equivalent to position 384 in TIA1, was previously generated [6,11,38]. The other plasmids with amino acid modifications of the TIA1 and TIAR proteins were generated by *around-the-horn PCR* site-directed mutagenesis using the corresponding plasmid templates [11]. The antisense and sense oligos used were synthesised by IDT Technologies and are detailed in Table S1 (Supplementary Materials).

After the corresponding PCR reactions, the products were purified (Wizard^®^ SV Gel and PCR Clean-Up System from Promega, Madison, WI, USA) and treated with 1 μl Dpnl enzyme (BioLabs, Ipswich, MA, USA) for 15 min at 37 °C to remove the DNA template. Then, the plasmids were ligated with T4 DNA ligase (BioLabs, Ipswich, MA, USA) for 30 min at 37 °C and transformed into competent *Escherichia coli* DH5a by heat shock for 1.5 min at 42 °C. The product of each transformation was cultured in Petri dishes with Luria–Bertani (LB) agar supplemented with 50 μg/mL of ampicillin at 37 °C overnight. Four colonies were selected from each plate and cultured for 16–18 h at 37 °C in 4 mL of LB medium supplemented with 50 μg/mL of ampicillin. The corresponding plasmids were purified from liquid cultures (Wizard^®^ Plus SV Minipreps DNA Purification System kit from Promega). The sequence of the purified plasmids was verified by automated DNA sequencing (Macrogen, Madrid, Spain).

### 2.3. Transfections and Oxidative Stress Induction

HEK-Flp cells were transfected, at 50% confluence, with 1.5 µg of the corresponding plasmid using TurboFect (Thermo Scientific, Waltham, MA, USA) according to the manufacturer’s instructions. After 2–4 h, protein expression was induced by adding tetracycline (100 ng/mL; Merck, Darmstadt, Germany) for 24 h. To induce oxidative stress, sodium arsenite (0.5 mM; Merck, Darmstadt, Germany) was added 24 h after the tetracycline, and cells were collected for analysis after 1 h of incubation [38,39].

### 2.4. Western Blotting Analysis

Cells were cultured and processed for Western blot analysis as previously described [3,14,26,27] using the following antibodies (all from Santa Cruz Biotechnology, Dallas, TX, USA): anti-TIA1 (sc-1751; 1/3000), anti-HuR (3A2) (sc-5261; 1/4000), and anti-TIAR (sc-1749; 1/3000). Secondary antibodies were conjugated with Horse Radish Peroxidase (HRP) (Promega, Madison, WI, USA). Membranes were developed with ECL reagent (Amersham, Amersham, UK) following the manufacturer’s instructions. Western blot bands were quantified with ImageJ/Fiji-win64 1.54f software.

### 2.5. Immunofluorescence and Confocal Microscopy

For confocal microscopy analysis, HEK293-Flp cells were cultured on coverslips, transfected, and fixed for 15–20 min with 10% formalin (Merck, Darmstadt, Germany). An anti-G3BP1 antibody (CSB-PA009116GA01HU, Cusabio Technology LLC, Houston, TX, USA) was used to label SGs and To-Pro3 (1 µM) (Invitrogen, Waltham, MA, USA) was used for selective staining of the nucleus. ImageJ software was used for image processing. The images were visualised and recorded using the 60x objective of a Laser Scanning Confocal Microscope A1R + coupled to an inverted Eclipse Ti-E microscope (Nikon, Tokyo, Japan) [6,11,38,39].

### 2.6. Other Informatics Resources

The genetic map of TIA1 and information regarding its exons and organisation were obtained from NCBI, making modifications with the SnapGene Viewer 8.0 software. The 3D structures of the TIA1 and TIAR proteins were obtained from the AlphaFold database (https://alphafold.ebi.ac.uk/; accessed on 21 November 2024) [40,41]. The I-TASSER server is an online platform that implements I-TASSER-based algorithms for protein structure and function predictions (http://zhanglab.ccmb.med.umich.edu/I-TASSER; accessed on 21 November 2024). It allows academic users to automatically generate high-quality model predictions of the 3D structure and biological function of protein molecules from their amino acid composition [42].

## 3. Results

### 3.1. TIA1-Mutant-Dependent SG Assembly Analysis: The Importance of Residue 384

**Figure 1:**
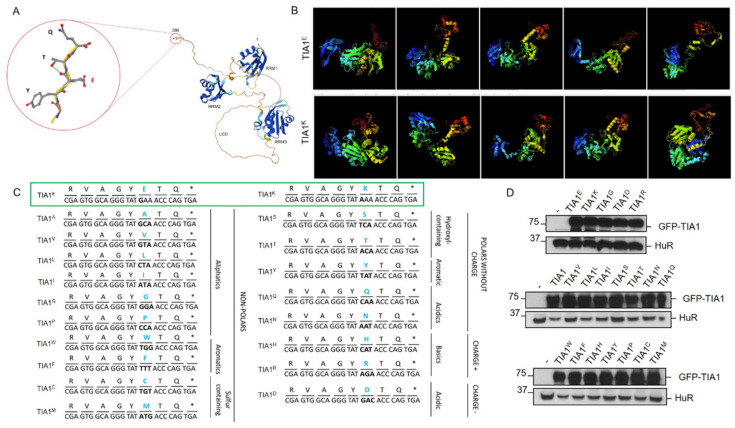
Collection of amino acid variants at position 384 of human TIA1. (**A**) Schematic 3D structure of the human TIA1 protein with the in silico AlphaFold tool. The image shows the first and the last residues, the RRMs 1-3, and the C-terminal LCD of human TIA1. The encircled area indicates a C-terminal extension with the amino acid at position 384 of TIA1 highlighted in red. (**B**) Images of automated protein structure prediction and structure-based function annotation of structural models of WT and WDM TIA1 variants. The image series illustrates the WT (upper pictures) and WDM (lower pictures) 3D TIA1 structural models for the top five options estimated by the in silico iTASSER tool [43]. Green and blue structures indicate the RRM 1-3 and the yellow/red one shows the LCD terminal domain. (**C**) Generation of the entire collection of TIA1 mutants dependent on residue 384. Schematic of the sequence of the last eight amino acid residues of the human TIA1 protein with each of the substitutions (blue bold type), nucleotide triplets (highlighting in bold those containing the substitution/mutation), and the families or categories into which the TIA1 mutants were grouped. The asterisk identifies the nonsense or stop codon. The controls (WT/E) and mutant (WDM/K) are boxed in green. (**D**) Expression analysis of TIA1 mutants using the Western blot technique. Western blot of protein extracts from HEK-Flp cells transfected with the entire collection of GFP-TIA1 mutants at position 384 with the corresponding substitutions. The monoclonal antibodies used were anti-TIA1, with anti-HuR as a loading control marker. Molecular weight markers (kDa) and proteins identified are indicated on the left and right, respectively.

To illustrate the WDM-associated mutation location and protein structure of the human isoform TIA1, we used the AlphaFold tool (Figure 1A). Thus, we could observe the three RRMs and LCD motifs of TIA1, together with the WDM-associated substitution of a glutamic acid residue (E) for lysine (K). The results of the I-TASSER-based algorithms for protein structure and function predictions can be seen in Figure 1B and Figure S1. To further study the role of amino acids at position 384 of human TIA1 in SG formation dynamics, the original glutamic acid (Glu/E) was replaced—by lysine (Lys/K), in the case of WDM—by each of the proteinogenic amino acids. For this, oligonucleotides with specific triplets corresponding to each amino acid were designed (Figure 1C). These substitutions generated asparagine (Asn/N), glutamine (Gln/Q), alanine (Ala/A), valine (Val/V), tyrosine (Tyr/Y), histidine (His/H), proline (Pro/P), methionine (Met/M), serine (Ser/S), tryptophan (Trp/W), cysteine (Cys/C), leucine (Leu/L), isoleucine (Ile/I), phenylalanine (Phe/F), and threonine (Thr/T) residues. These oligonucleotides were used to perform a PCR approach called “round-the-horn PCR” (Annex II), which allowed for the complete copying of pcDNA-5/FRT/TO plasmids with the GFP-TIA1 protein containing each of the desired substitutions/mutations (Figure 1D). The amplicons were ligated and transformed for inoculation into liquid cultures of four independent colonies of each of the constructs to isolate plasmid DNA and verify the substitutions by automated DNA sequencing. Subsequently, all TIA1 variants were transiently transfected into HEK293-Flp cells for Western blot analysis and to verify the degree of expression of the corresponding GFP-TIA1 fusion proteins. As a loading control, the endogenous expression of HuR was determined (Figure 1D). The results obtained show that all constructs analysed were efficiently transfected and produced similar amounts of each GFP-TIA1 fusion protein variant, with comparable protein steady states, suggesting that protein stability it is not compromised between the different variants of TIA1 (Figure 1D).

For confocal microscopy fluorescence analyses, the GFP-TIA1 mutant versions of the 20 proteinogenic amino acids (Figure 1C, green boxes) were used. As controls and references for the formation of TIA1-dependent SGs, the plasmids were also transfected with WT TIA1 (E384) and TIA1 containing the WDM mutation (E384K) (Figure 1C, green box).

**Figure 2:**
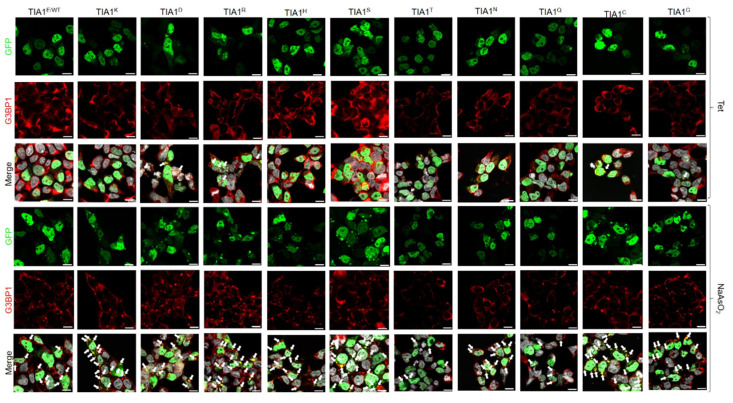
Differential dynamics of stress granules dependent on TIA1 variants at position 384. Fluorescence microscopy images of HEK-Flp cells transfected with GFP-TIA1 plasmids and their variants, identified in the legend at the top of this panel. Expression of the GFP-TIA1 fusion protein is shown in green, the G3BP1 antibody in red, and “Merge” corresponds to the combination of the three channels: GFP (green), G3BP1 (red), and To-Pro3 (grey-stained nuclei). The white arrows indicate TIA1-SGs. Images are shown both in the absence and presence of sodium arsenite treatment for each mutation examined. Scale bar, 10 μm.

**Figure 3:**
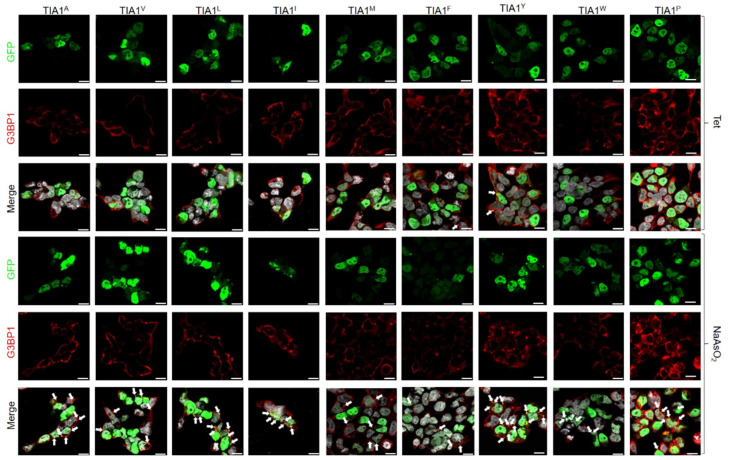
Alternative dynamics of stress granules dependent on TIA1 variants at position 384. Fluorescence microscopy images of HEK-Flp cells transfected with GFP-TIA1 plasmids and their variants, identified in the legend at the top of this panel. Expression of the GFP-TIA1 fusion protein is shown in green, the G3BP1 antibody in red, and “Merge” corresponds to a combination of the three channels: GFP (green), G3BP1 (red), and To-Pro3 (grey-stained nuclei). The white arrows illustrate TIA1-SGs. Images are shown both in the absence and presence of sodium arsenite treatment for each mutation examined. Scale bar, 10 μm.

Previous studies demonstrated that the expression of the TIA1^E384K^ mutated protein altered the size and quantity of SGs [11,24,39]. Therefore, the same parameters were analysed with confocal microscopy in HEK293 cells transfected with all the plasmid constructs generated. The first study focused on spontaneous SG assembly followed by oxidative stress-induced SG formation using 0.5 mM sodium arsenite for 1 h (Figure 2 and Figure 3).

In this sense, results confirmed that the subcellular location of GFP-TIA1 variants was that expected for the endogenous protein, i.e., nucleocytoplasmic with nucleolar exclusion [11,38,39] (Figure 2 and Figure 3). The differences observed in signal intensities may derive from the different expression of GFP between cells as a result of transfection efficiency (20–30%) and the cell culture’s growth dynamics. Nevertheless, for all variants, SG formation was observed in the transfected cells under oxidative stress (Figure 2 and Figure 3).

**Figure 4:**
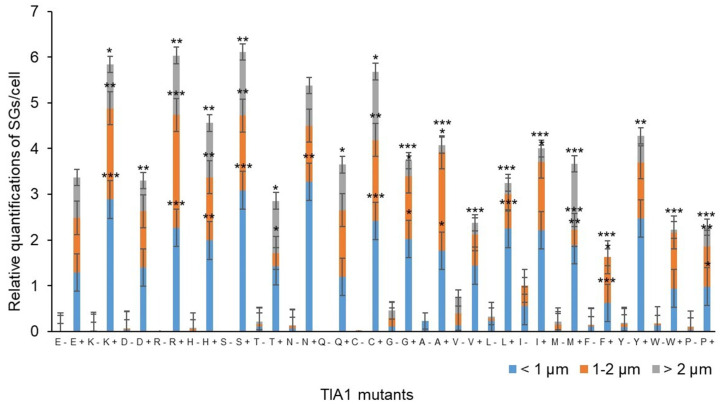
Quantification of the relative number and size of TIA1 variant-dependent SGs described in Figure 2 and Figure 3. Data represent the mean ± standard error of the mean (n = 18–114 cells for each condition; * *p* < 0.5; ** *p* < 0.1; *** *p* < 0.05).

Afterwards, based on the images taken, the total number of SGs formed per cell was calculated for each variant. Also, SG size was measured, and they were classified into three categories: <1 μm, between 1 and 2 μm, and >2 μm (Figure 4). Comparing the results with those from the TIA1^E^ sample suggests that introducing amino acids with a positively charged side chain—arginine (R), histidine (H), and lysine (K, associated with WDM)—at position 384 of TIA1 (the predominant isoform in the cell type studied), instead of the WT glutamic acid (E) residue, favours the formation of more TIA1 SGs under oxidative stress conditions. The most striking case was that of the R residue, which showed a significant gain in all three TIA1 SG size categories. The relative gain regarding SG formation for this amino acid category would be K^WDM^ = R ≥ H > E^WT^. On the other hand, the presence of an amino acid with a negatively charged side chain, i.e., aspartic acid (D), did not present any significant differences to the E residue, with even fewer granules of the largest size (>2 μm). Thus, the relative gain or loss with respect to SG formation for this amino acid category compared with the WDM variant would be K^WDM^ > E^WT^ = D (Figure 2 and Figure 4).

Concerning the behaviour associated with amino acids with uncharged polar side chains, the situation can be defined as heterogeneous. For example, the introduction of a serine (S) residue promoted an increase in SG number, with similar parameters to those observed with positively charged amino acids, particularly favouring the formation of small-sized granules. This contrasts with the observations for the threonine residue (T), which did not favour an increase in granule number but did result in more granules of larger size. However, glutamine (Q) reproduced the situation observed with the WT (E), albeit with some larger-sized granules. However, this was not seen for asparagine (N), which increased the number of small granules, in a situation more similar to that seen with WDM and positively charged amino acids. Therefore, the relative gain or loss concerning SG formation for this amino acid category compared with WDM and WT would be K^WDM^ = S ≥ N > Q = E^WT^ ≥ T (Figure 2 and Figure 4).

In the same vein, the amino acids considered “special cases” due to the nature of their side chains—cysteine (C) with a sulfhydryl or thiol group, glycine (G) with hydrogen, and proline (P), an amino acid which does not contain the amino group (-NH_2_)—the result was also heterogeneous. The presence of C reproduced the situation of positively charged amino acids, with an increase in SG number and size; the presence of G showed similar results to those observed with WT; and the P residue very significantly reduced both SG number and size. Therefore, the relative gain or loss concerning SG formation for this amino acid category compared with WDM and WT would be K^WDM^ = C > G = E^WT^ > P (Figure 3 and Figure 4).

Regarding amino acids with hydrophobic side chains, their most striking feature was to reproduce situations of heterogeneity, such as those observed in amino acids with uncharged polar side chains. Thus, the presence of a tyrosine (Y) residue increased the number of SGs, especially those of smaller size, but without reaching the levels observed with C and S residues. On the other hand, alanine (A), methionine (M), isoleucine (I), and leucine (L) residues exhibited a similar situation, with small variations between size categories, to that observed in WT. However, in the presence of phenylalanine (F) and tryptophan (W) residues, the reduction in the number and size of granules was significant, being even more evident with valine (V), which resembled the situation observed with P. Consequently, the relative gain or loss concerning SG formation with this amino acid category compared with WDM and WT would be K^WDM^ > Y > A = M = E^WT^ = I = L > F = W > V (Figure 3 and Figure 4).

TIA1^Y^ displays an important capacity for spontaneous SG formation, like TIA1^H^, TIA1^N^, and TIA1^C^, but in a more moderate manner. SGs generated in the absence of stressful stimuli were mostly smaller (<1 μm), although with some granules between 1 and 2 μm (Figure 2 and Figure 4).

The treatment of cells with sodium arsenite caused the appearance of numerous SGs in all samples; however, SGs from TIA1^K^ and TIA1^H^ were more prominent compared with TIA1^E^ and were also preferentially of small-medium size (Figure 2 and Figure 4). In addition, the expression of variants TIA1^N^, TIA1^Y^, TIA1^S^, and TIA1^C^ did not show a significant increase in SG number compared to TIA1^E^, and their granules were predominantly of medium-small size. Thus, the presence of the amino acids asparagine, tyrosine, serine, and cysteine at position 384 of the TIA1 protein does not seem to cause changes in the formation–assembly dynamics of TIA1-dependent SGs.

However, the expression of the TIA1^P^ variant is related to a decrease in SGs compared with TIA1^E^. At the opposite extreme is TIA1^H^, the variant that presents fewer SGs of medium-high size and more of small size. The remaining mutants generated a percentage of large-sized granules similar to that observed with TIA1^K^ (Figure 2, Figure 3 and Figure 4).

### 3.2. TIAR-Mutant-Dependent SG Formation Analysis: Is the Nature of Residue 373 Important?

**Figure 5:**
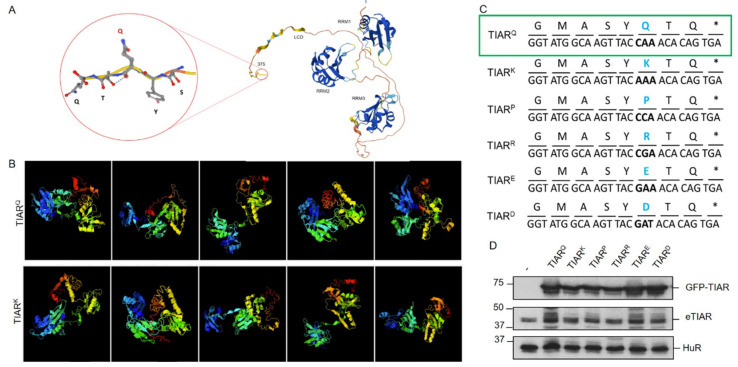
Collection of amino acid variants at position 373 of human TIAR. (**A**) Schematic 3D structure of the human TIAR protein by the in silico AlphaFold tool. The image shows the first and last residues, the RRMs 1–3, and the C-terminal LCD of human TIAR. The encircled area indicates a C-terminal extension with the amino acid located at position 373 in TIAR highlighted in red. (**B**) Images of automated protein structure prediction and structure-based function annotation of structural models of TIAR^Q^ (WT) and TIAR^K^ variants. The image series illustrates the WT (upper pictures) and TIAR^K^ (lower pictures) 3D TIAR structural models for the top five options estimated by the in silico iTASSER tool. Green and blue structures indicate the RRM 1-3 and the yellow/red one shows the LCD terminal domain. (**C**) Generation of the collection of TIAR mutants dependent on Q residue. Schematic of the sequence of the last eight amino acid residues of the human TIAR protein with each of the substitutions (blue bold type), and nucleotide triplets (highlighting in bold those containing the substitution/mutation). The asterisk identifies the nonsense or stop codon. Controls (WT/TIAR^Q^) are boxed in green. (**D**) Expression analysis of TIAR mutants using the Western blot technique. Western blot of protein extracts from HEK-Flp cells transfected with the entire collection of GFP-TIAR mutants with the corresponding substitutions. The monoclonal antibodies used were anti-TIA1, with anti-HuR used as a loading control marker. Molecular weight markers (kDa) and proteins identified are indicated on the left and right, respectively.

For a better understanding of the molecular function of TIA1 in SG formation associated with WDM, the behaviour of TIAR/TIAL1, as the TIA1 paralog, was also studied. The TIAR/TIAL1 protein shares a high structural conservation and identity in its RRM domains with TIA, although the LCD differs by about 50% [14] (Figure 5A,B). 

The results of the I-TASSER-based algorithms for TIAR protein structure and function predictions can be seen in Figure 5B and Figure S2. TIAR also plays a complementary biological role to that of TIA1, forming part of SGs [2,25]. Therefore, an equivalent mutagenesis strategy was performed to introduce several amino acid residues in TIAR, equivalent to position 384 of TIA1, which were previously shown to affect TIA1-dependent SG formation/assembly [12,22]. In this sense, the same experimental approaches previously described for TIA1 were applied (Figure 5C).

**Figure 6:**
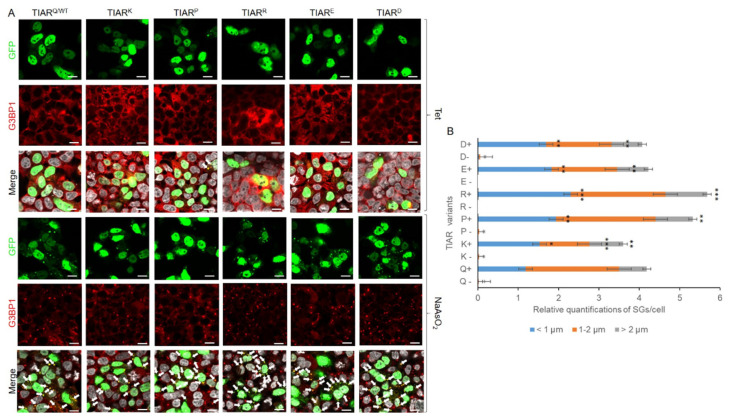
Differential dynamics of stress granules dependent on TIAR variants. (**A**) Fluorescence microscopy images of HEK-Flp cells transfected with GFP-TIAR and their variants, identified in the legend at the top of this panel. Expression and distribution/location of the GFP-TIAR fusion protein are shown in green, the G3BP1 antibody in red, and “Merge” corresponds to the combination of the three channels: GFP, G3BP1, and To-Pro3 (grey-stained nuclei). The white arrows show TIAR-SGs. Images are shown both in the absence and presence of sodium arsenite treatment for each mutation studied. Scale bar, 10 μm. (**B**) Quantification of SG number and size under the experimental described in (**A**). Data represent the mean ± standard error of the mean (n = 10–30 cells for each condition; * *p* < 0.5; ** *p* < 0.1; *** *p* < 0.05).

The first amino acids chosen were lysine (K), as it is the amino acid associated with WDM, and glutamic acid (E), which appears in WT TIA1. The other amino acids selected were proline (P), arginine (R), and aspartic acid (D). As mentioned, the experimental protocol was the same as for TIA1, using “*round-the-horn PCR*” to obtain pcDNA-5/FRT/TO-GFP-TIAR with the desired mutations (Figure 5C) (Annex III). Western blot analysis for the verification of fusion GFP-TIAR protein expression (Figure 5D) indicated that all constructions were feasible and generated measurable and homogeneous quantities of the different GFP-TIAR variants. Additionally, immunofluorescence analysis was conducted to visualise TIAR-dependent SGs. Parameters and criteria for SG quantification dependent on TIAR variants were the same as for TIA1. In all cases, the increase in SG formation was observed after treatment with sodium arsenite, and differences in GFP intensity may also be due to the transfection efficiency (approx. 20–30%) and dynamics of the cell culture itself (Figure 6).

TIAR is ubiquitously expressed and conserved among different mammalian species [12,22]. A key finding from the mutagenic analysis of TIAR at the glutamine residue—equivalent to the glutamic acid residue of TIA1, which is mutated to lysine in WDM—is that the change to lysine had no positive effect on the formation of TIAR SGs (Figure 6). The same was observed when negatively charged amino acids, such as aspartic (D) or glutamic acid (E), were introduced. However, the introduction of an arginine (R) slightly raised the number of granules per cell and within each of the relative granule size categories.

Thus, analysis of the total number of SGs per cell indicated that TIAR^P^ is the only variant that presents spontaneous SG formation, including TIAR^Q^; these granules were mainly medium-sized (between 1 and 2 μm) (Figure 6A,B). When treated with sodium arsenite, a large number of SGs appeared in all samples, highlighting TIAR^Q^, TIAR^R^, TIAR^E^, and TIAR^D^. Almost all the SGs generated in these samples were of small-medium size, without any large granules, except in TIAR^R^ and TIAR^P^, where a small but significant presence of granules > 2 μm was observed. On the contrary, small-medium-sized granules were abundant in all samples, including those with large granules. TIAR^K^ generates a lower number of granules in the stress situation, even less than WT TIAR^Q^. Consequently, these observations suggest that lysine exerts opposing effects in TIAR and TIA1 and is associated with WDM.

On the other hand, it was unexpected that the introduction of proline (P) would also slightly increase the number of TIAR SGs, the result being similar to that obtained with the R residue. Collectively, these results (i) show that the WDM-associated *TIA1* mutation only has consequences in the amino acid context of TIA1 and not in its paralog TIAR; (ii) reinforce the relevant role of R residues in the dynamics and molecular grammar of SG formation, independently of RBPs with IDR domains, as their occurrence has been demonstrated in TIA1 (this and previous studies), TIAR (this study), and FUS [23]; and (iii) the unexpected role of proline with antagonistic effects in proteins with highly conserved primary amino acid sequences. Thus, the relative gain concerning TIAR-dependent SG formation for the amino acid categories tested compared with that observed with WT TIAR would be R ≥ P > Q^WT^ = K = E = D (Figure 6). In short, the molecular grammar of TIA1 and TIAR SGs shows neutral, shared/suppressing and/or potentiating, and even opposing/antagonistic and/or negative dynamics associated with the behaviour of specific amino acid residues.

## 4. Discussion

TIA1-dependent SGs are molecular condensates that appear in the cytoplasm of environmentally stressed cells and whose assembly is caused by a disruption of translation initiation [2,25,28]. Previous studies have shown that the TIA1^K^ variant (present in WDM) is associated with a dysfunction in SG formation. The existence of these SGs in the cell has an important role in physiological conditions. In this way, SGs have been proposed as signalling modulators and enhancers of the formation of translation initiation complexes. However, the anomalous appearance or increased stability of these SGs can cause cell death by changes in cell homeostasis, such as altered regulation of RNA biogenesis and function, the dysregulation of signalling cascades, and defects in nucleus-cytoplasm RNA transport [11,24,28,33,43]. For this reason, in vivo models are essential to study the molecular grammar of TIA1.

**Figure 7:**
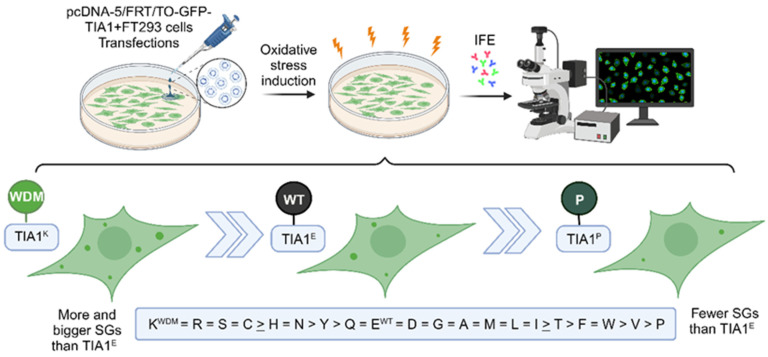
Summary of the impact of TIA1 variants at position 384 on the dynamics of TIA1-dependent stress granules. WT represents wild-type TIA1, WDM illustrates TIA1 containing the p.E384K mutation associated with Welander distal myopathy, and P indicates proline residue-associated behaviour, where the number of SGs is much lower, in transfected FT293 cells with TIA1 variants under oxidative stress conditions (sodium arsenite). This figure was created with BioRender.com. [47].

In this study, we addressed the mutagenic analysis of the glutamic acid (E/WT) residue at position 384 of human isoform TIA1a. This allowed us to elucidate the potential and incidence of 20 proteinogenic amino acids on the formation of TIA1-dependent SGs under oxidative stress conditions induced by sodium arsenite. We identified the amino acid residues that most favour the formation of TIA1 SGs and those that most hinder it, taking as references the glutamic residue (E) in the WT variant and the lysine (K) residue in the mutated version of human TIA1 associated with WDM. Based on the results obtained, the order would be as follows: K^WDM^ = R = S = C ≥ H = N > Y > Q = E^WT^ = D = G = A = M = L = I ≥ T > F = W > V > P (Figure 7). These observations support our hypothesis that certain amino acids exhibit a more prevalent molecular grammar than others, even though they belong to different amino acid families. However, above all, there is a family of amino acids with electrically charged side chains that exhibit consistent behaviour, albeit in two directions. This family includes positively charged amino acids (R, K, and H) that favour the formation and dynamics of TIA1 SGs, in contrast to negatively charged amino acids (E and D), which serve as references and represent the homeostatic condition.

TIA1^E^-transfected cells generated fewer SGs under stress conditions than those transfected with TIA1^R^ or TIA1^H^, as expected from previous assays performed with TIA1^K^ and TIA1^R^ [11,24,39]. However, it is striking that there were no differences in the amount or size of the SGs generated between the two variants, TIA1^K^ and TIA1^H^, which suggests that it is the presence of a positive charge in this region, rather than the intensity of the charge [38], that alters SG dynamics. In the same vein, it could be suggested that SGs created by positively charged variants (lysine, arginine, and histidine) [11,39] would be more persistent over time than those generated by TIA1^WT^ or any other variant analysed in this and previous studies. This would imply that the presence of a positive charge favours the interaction and, perhaps, the stable aggregation of the protein [11,24,39].

The other TIA1 variants studied exhibited the same behaviour as TIA1^WT^ in terms of SG number and size, signifying that simply eliminating the negative charge of glutamic acid is not sufficient to alter protein behaviour or SG formation; it must be replaced by a positive charge or proline for this change to occur. Another significant result was that the presence of proline at position 384 after the addition of sodium arsenite resulted in significantly fewer SGs, indicating that this amino acid may hinder, to some extent, the protein’s ability to form condensates. In this sense, it was recently demonstrated that proline-rich domains affect the LLPS processing of proteins such as UBQLN2 and Tau. Therefore, proline is involved in the regulation of LLPS processing [44,45]. The LCD of TIA1 has 11 proline residues, and it has been experimentally demonstrated that substitution of these prolines facilitates the transition from TIA1 to LLPS, while proline substitution delays the intracellular disassembly of SGs. In addition, proline-associated mutations in TIA1 prevent TIA1-dependent SG clearance. Our results indicate that the introduction of a proline residue decreases the formation and/or facilitates the clearance of TIA1-dependent SGs, which agrees with the relevant role of proline in TIA1-dependent SG dynamics [45].

Also, the molecular grammar—defined as the driving force underlying or governing phase separation and the formation of liquid condensates, which can harden into less-liquid structures depending on amino acid composition and their characteristics—was coined in the study of FUS family dynamics [32]. This work highlighted the relevance of multivalent interactions between tyrosine residues in prion-like domains (PLDs) and arginine residues of RRMs. They identified that glycine residues increased fluidity, whereas glutamine and serine residues favoured stiffening. Thus, their results suggested that it was possible to predict the fluidity or condensation properties of proteins with prion-like domains based on amino acid sequences. Our experimental observations of the prion-like protein TIA1 demonstrate the relevant role played in TIA1-dependent SG formation by K, R, H, Y, N, C, and S residues, as well as A and G for fluidity gain. On the other hand, our results also indicate a role for cysteine, probably because of its ability to form disulfide bridges in an oxidative environment and favour intramolecular interaction as well as the prevalent role associated with post-translational modifications, such as phosphorylation linked to tyrosine and serine residues.

In the same vein, recent evidence has emerged suggesting that many RBPs containing IDRs lead to self-recruitment in the cellular context [46]. This has allowed their classification by grouping RBPs according to the characteristics of their IDRs, namely: amino acid composition, hydrophobicity, charge, and tendency to disorder; thus, five clusters have been established. For example, TIA1 and TIAR have been assigned to “Cluster 1”, characterised by having a constant charge or uncharged IDRs, with a tendency to disorder, and a hydrophobicity associated with their IDR region. Interestingly, most of the RBPs assigned to this group are enriched in SGs and paraspeckles. In contrast, RBPs from Cluster 3 are enriched in nuclear speckles. These observations in the context of SGs could also have an impact on the RNAs recruited to SGs. Thus, RBPs could present charged or uncharged IDRs that impact multivalent interactions such as the cleavage or mixing of the components involved in LLPS. Given these observations, we suggest that the absence or presence of charges in the IDR of TIA1 may modify the interactions of TIA1-dependent SGs with WDM mutations. This also extends to other residues that contribute opposite charges, as well as to residues susceptible to phosphorylation (i.e., Y, S, and T), which can change their charge due to potential post-translational modifications. Additionally, these modifications can affect their hydrophobicity and solubility.

Overall, the results agree with and expand on those obtained by Carrascoso et al. (2019) [11] and Fernández-Gómez et al. (2022) [39], who generated TIA1 variants by substituting only the amino acid mutated in WDM with other amino acids with different charges: one or three arginines (positive charge), one or three aspartic acids (negative charge), and one or three alanines (uncharged). They also found that positive charges favour SG formation more than neutral charges, which in turn favour SG formation to a greater extent than negative charges.

Finally, we also designed several variants of the TIA1 paralog, known as TIAR/TIAL1, and observed that this situation did not cause the same substantial impact on the kinetics of TIAR-dependent SG formation. In this sense, the results show that TIAR is sensitive to the presence of arginine at that position, which has opposite and negative effects on TIAR SG dynamics. The same was true for proline, with opposite effects to those observed with TIA1. This fact highlights that certain molecular and cellular events during the LLPS process are shared and could even be universal principles. However, there are specific and differential details between the two proteins that would not even be extrapolatable using the bioinformatics tools developed from the previous studies [44,46]. The limitations of current study are associated with the ectopic overexpression of the transgenes, since may cause a gain-of-function effect that does not accurately reflect the endogenous situation. To address these limitations, more controlled approaches, such as expressing the transgene in a TIA1-null background or using a knock-in strategy (endogenous gene editing) may be ideal. However, transgenes such as those used in this study have been previously used to reproduce the endogenous nucleo-cytoplasmic patterns of TIA1 in the same cell type at short time (24 h post-induction) under conditions of homeostasis and oxidative stress [6]. Anyway, further investigations are required to unravel the secrets of the multivariate interactions responsible for the biophysical phenomenon of LLPS.

## 5. Conclusions

As a practical conclusion, all mutants or substitutions can be generated, transfected, and examined for their in vivo expression to expand the set of TIA1 variants to the 20 proteinogenic amino acids at position 384 associated with WDM. Notably, the TIA1^R^ variant mimics the effects of the WDM mutation, while the TIA1^P^ variant produces antagonistic effects. Interestingly, translating these substitutions to the TIAR paralog reveals a contrary situation, as evidenced by observing the reduction in SG number or the absence of effects associated with either negative or positive charges. The introduction of arginine and proline residues in TIAR produced results contrary to those observed in TIA1. This has broadened our existing knowledge of how the TIA1 WDM mutation (E384K) impacts cellular homeostasis, particularly TIA-SG dynamics, while shedding light on the role of the protein’s C-terminal domain and how its “molecular grammar” affects this biological phenomenon.

## Data Availability

The original contributions presented in the study are included in the article/Supplementary Materials, further inquiries can be directed to the corresponding author.

## Supplementary Materials

The following supporting information can be downloaded at: https://www.mdpi.com/article/10.3390/cells13231961/s1, Figure S1. iTASSER analysis of TIA1 variants used in this study. Figure S2. iTASSER analysis of TIAR variants used in this study. Figure S3. Original Western blot analysis images. Table S1. Sequences of oligonucleotides used in this study.

## References

[1] Tian Q., Streuli M., Saito H., Schlossman S.F., Anderson P. (1991). A polyadenylate binding protein localized to the granules of cytolytic lymphocytes induces DNA fragmentation in target cells. Cell.

[2] Anderson P., Kedersha N. (2002). Stressful initiations. J. Cell Sci..

[3] López de Silanes I., Galbán S., Martindale J.L., Yang X., Mazan-Mamczarz K., Indig F.E., Falco G., Zhan M., Gorospe M. (2005). Identification and functional outcome of mRNAs associated with RNA-binding protein TIA-1. Mol. Cell. Biol..

[4] Reyes R., Alcalde J., Izquierdo J.M. (2009). Depletion of T-cell intracellular antigen proteins promotes cell proliferation. Genome Biol..

[5] Wang Z., Kayikci M., Briese M., Zarnack K., Luscombe N.M., Rot G., Zupan B., Curk T., Ule J. (2010). iCLIP predicts the dual splicing effects of TIA-RNA interactions. PLoS Biol..

[6] Sánchez-Jiménez C., Ludeña M.D., Izquierdo J.M. (2015). T-cell intracellular antigens function as tumor suppressor genes. Cell Death Dis..

[7] Van Nostrand E.L., Freese P., Pratt G.A., Wang X., Wei X., Xiao R., Blue S.M., Chen J.Y., Cody N.A.L., Dominguez D. (2020). A large-scale binding and functional map of human RNA-binding proteins. Nature.

[8] Horste E.L., Fansler M.M., Cai T., Chen X., Mitschka S., Zhen G., Lee F.C.Y., Ule J., Mayr C. (2023). Subcytoplasmic location of translation controls protein output. Mol. Cell.

[9] Cho N.H., Cheveralls K.C., Brunner A.D., Kim K., Michaelis A.C., Raghavan P., Kobayashi H., Savy L., Li J.Y., Canaj H. (2022). OpenCell: Endogenous tagging for the cartography of human cellular organization. Science.

[10] The Human Protein Atlas (2022): TIA1. https://www.proteinatlas.org/ENSG00000116001-TIA1.

[11] Carrascoso I., Sánchez-Jiménez C., Silion E., Alcalde J., Izquierdo J.M. (2019). A heterologous cell model for studying the role of T-cell intracellular antigen 1 in Welander distal myopathy. Mol. Cell Biol..

[12] Fernández-Gómez A., Izquierdo J.M. (2022). The multifunctional faces of T-cell intracellular antigen 1 in health and disease. Int. J. Mol. Sci..

[13] Kawakami A., Tian Q., Streuli M., Poe M., Edelhoff S., Disteche C.M., Anderson P. (1994). Intron-exon organization and chromosomal localization of the human TIA-1 gene. J. Immunol..

[14] Beck A.R., Medley Q.G., O’Brien S., Anderson P., Streuli M. (1996). Structure, tissue distribution and genomic organization of the murine RRM-type RNA binding proteins TIA-1 and TIAR. Nucleic Acids Res..

[15] Izquierdo J.M., Valcárcel J. (2007). Two isoforms of the T-cell intracellular antigen 1 (TIA-1) splicing factor display distinct splicing regulation activities. Control of TIA-1 isoform ratio by TIA-1-related protein. J. Biol. Chem..

[16] Dember L.M., Kim N.D., Liu K.Q., Anderson P. (1996). Individual RNA recognition motifs of TIA-1 and TIAR have different RNA binding specificities. J. Biol. Chem..

[17] Wang I., Hennig J., Jagtap P.K.A., Sonntag M., Valcárcel J., Sattler M. (2014). Structure, dynamics and RNA binding of the multi-domain splicing factor TIA-1. Nucleic Acids Res..

[18] Kawakami A., Tian Q., Duan X., Streuli M., Schlossman S.F., Anderson P. (1992). Identification and functional characterization of a TIA-1-related nucleolysin. Proc. Natl. Acad. Sci. USA.

[19] Mazan-Mamczarz K., Lal A., Martindale J.L., Kawai T., Gorospe M. (2006). Translational repression by RNA-binding protein TIAR. Mol. Cell. Biol..

[20] Kim H.S., Kuwano Y., Zhan M., Pullmann R., Mazan-Mamczarz K., Li H., Kedersha N., Anderson P., Wilce M.C., Gorospe M. (2007). Elucidation of a C-rich signature motif in target mRNAs of RNA-binding protein TIAR. Mol. Cell. Biol..

[21] Kim H.S., Headey S.J., Yoga Y.M., Scanlon M.J., Gorospe M., Wilce M.C., Wilce J.A. (2013). Distinct binding properties of TIAR RRMs and linker region. RNA Biol..

[22] Velasco B.R., Izquierdo J.M. (2022). T-cell intracellular antigen 1-like protein in physiology and pathology. Int. J. Mol. Sci..

[23] The Human Protein Atlas (2022): TIAL1. https://www.proteinatlas.org/ENSG00000151923-TIAL.

[24] Hackman P., Sarparanta J., Lehtinen S., Vihola A., Evilä A., Jonson P.H., Luque H., Kere J., Screen M., Chinnery P.F. (2013). Welander distal myopathy is caused by a mutation in the RNA-binding protein TIA1. Ann. Neurol..

[25] Kedersha N.L., Gupta M., Li W., Miller I., Anderson P. (1999). RNA-binding proteins TIA-1 and TIAR link the phosphorylation of eIF-2 alpha to the assembly of mammalian stress granules. J. Cell Biol..

[26] Gilks N., Kedersha N., Ayodele M., Shen L., Stoecklin G., Dember L.M., Anderson P. (2004). Stress granule assembly is mediated by prion-like aggregation of TIA-1. Mol. Biol. Cell.

[27] Rayman J.B., Kandel E.R. (2017). TIA-1 is a functional prion-like protein. Cold Spring Harb. Perspect. Biol..

[28] Protter D.S.W., Parker R. (2016). Principles and properties of stress granules. Trends Cell Biol..

[29] Riback J.A., Zhu L., Ferrolino M.C., Tolbert M., Mitrea D.M., Sanders D.W., Wei M.T., Kriwacki R.W., Brangwynne C.P. (2020). Composition-dependent thermodynamics of intracellular phase separation. Nature.

[30] Klosin A., Oltsch F., Harmon T., Honigmann A., Jülicher F., Hyman A.A., Zechner C. (2020). Phase separation provides a mechanism to reduce noise in cells. Science.

[31] Jain S., Wheeler J.R., Walters R.W., Agrawal A., Barsic A., Parker R. (2016). ATPase-modulated stress granules contain a diverse proteome and substructure. Cell.

[32] Wang J., Choi J.M., Holehouse A.S., Lee H.O., Zhang X., Jahnel M., Maaharana S., Lemaitre R., Pozniakovsky A., Drechsel D. (2018). A molecular grammar governing the driving forces for phase separation of prion-like RNA binding proteins. Cell.

[33] Fritzsching K.J., Yang Y., Pogue E.M., Rayman J.B., Kandel E.R., McDermott A.E. (2020). Micellar TIA1 with folded RNA binding domains as a model for reversible stress granule formation. Proc. Natl. Acad. Sci. USA.

[34] Welander L. (1951). Myopathia Distalis Tarda Hereditaria; 249 Examined Cases in 72 Pedigrees. Acta Med. Scand. Suppl..

[35] Borg K., Ählberg G., Anvret M., Edström L. (1998). Welander distal myopathy—An overview. Neuromuscul. Disord..

[36] Von Tell D., Somer H., Udd B., Edström L., Borg K., Åhlberg G. (2002). Welander distal myopathy outside the Swedish population: Phenotype and genotype. Neuromuscul. Disord..

[37] Klar J., Sobol M., Melberg A., Mäbert K., Ameur A., Johansson A.C., Feuk L., Entesarian M., Orlén H., Casar-Borota O. (2013). Welander distal myopathy caused by an ancient founder mutation in TIA1 associated with perturbed splicing. Hum. Mutat..

[38] Carrascoso I., Alcalde J., Sánchez-Jiménez C., González-Sánchez P., Izquierdo J.M. (2017). T-cell intracellular antigens and Hu antigen R antagonistically modulate mitochondrial activity and dynamics by regulating optic atrophy 1 gene expression. Mol. Cell. Biol..

[39] Fernández-Gómez A., Velasco B.R., Izquierdo J.M. (2022). Dynamics of T-cell intracellular antigen 1-dependent stress granules in proteostasis and Welander distal myopathy under oxidative stress. Cells.

[40] Jumper J., Evans R., Pritzel A., Green T., Figurnov M., Ronneberger O., Tunyasuvunakool K., Bates R., Žídek A., Potapenko A. (2021). Highly accurate protein structure prediction with AlphaFold. Nature.

[41] Varadi M., Bertoni D., Magana P., Paramval U., Pidruchna I., Radhakrishnan M., Tsenkov M., Nair S., Mirdita M., Yeo J. (2024). AlphaFold protein structure database in 2024: Providing structure coverage for over 214 million protein sequences. Nucleic Acids Res..

[42] Yang J., Zhang Y. (2015). I-TASSER server: New development for protein structure and function predictions. Nucleic Acids Res..

[43] Ramaswami M., Taylor J.P., Parker R. (2013). Altered ribostasis: RNA-protein granules in degenerative disorders. Cell.

[44] van Mierlo G., Jansen J.R.G., Wang J., Poser I., van Heeringen S.J., Vermeulen M. (2021). Predicting protein condensate formation using machine learning. Cell Rep..

[45] Ding X., Gu S., Xue S., Luo S.Z. (2021). Disease-associated mutations affect TIA1 phase separation and aggregation in a proline-dependent manner. Brain Res..

[46] Masuda A., Okamoto T., Kawachi T., Takeda J.I., Hamaguchi T., Ohno K. (2024). Blending and separating dynamics of RNA-binding proteins develop architectural splicing networks spreading throughout the nucleus. Mol. Cell.

[47] Izquierdo J.M. (2024). BioRender.com/g36g809. https://www.biorender.com/g36g809.

