# Supplementary material for: Decoding the Molecular Grammar of TIA1-Dependent Stress Granules in Proteostasis and Welander Distal Myopathy Under Oxidative Stress
Source: Cells. 2024 Nov 27;13(23):1961. doi: 10.3390/cells13231961 (PMC11640499; doi:10.3390/cells13231961)
Supplement: Supplementary file 1 [file cells-13-01961-s001.zip › Supplementary information.pdf]

Figure S1

GLUTAMIC ACID (Glu, E)

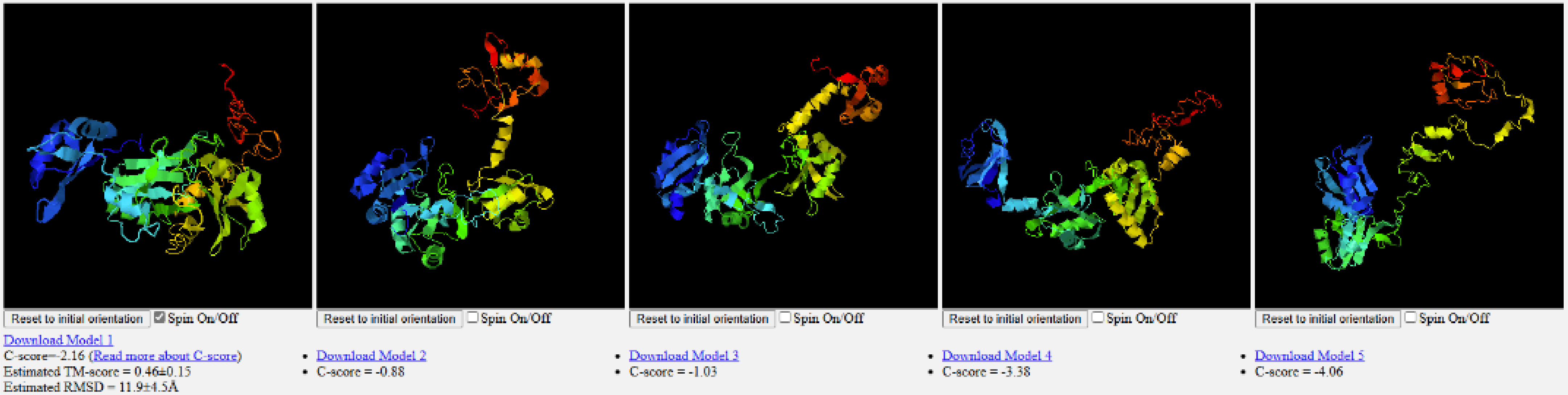

Reset to initial orientation | Spin On/Off (×5)

- Download Model 1
- C-score=-2.16 (Read more about C-score)
- Estimated TM-score = 0.46±0.15
- Estimated RMSD = 11.9±4.5Å

- Download Model 2
- C-score = -0.88

- Download Model 3
- C-score = -1.03

- Download Model 4
- C-score = -3.38

- Download Model 5
- C-score = -4.06

LYSINE (Lys, K)

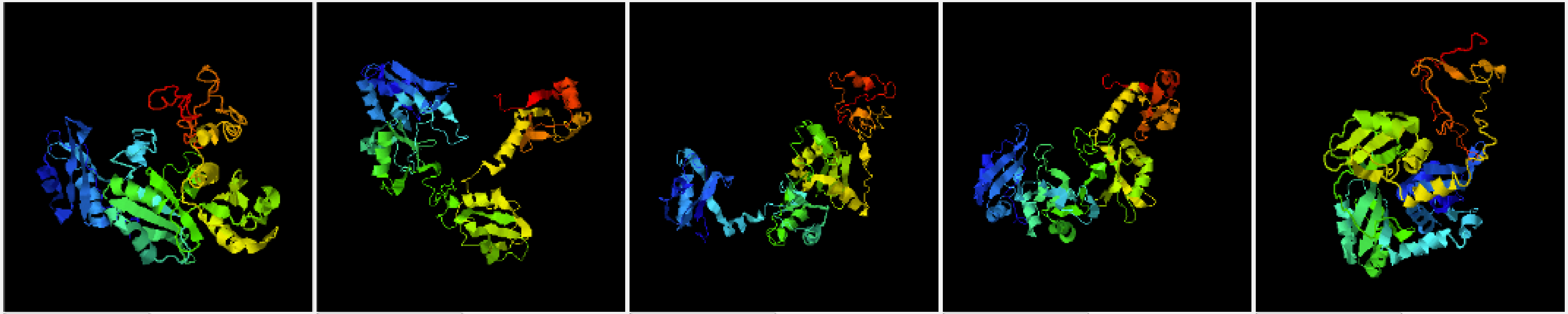

Reset to initial orientation | Spin On/Off (×5)

- Download Model 1
- C-score=-1.18 (Read more about C-score)
- Estimated TM-score = 0.57±0.15
- Estimated RMSD = 9.4±4.6Å

- Download Model 2
- C-score = -1.21

- Download Model 3
- C-score = -2.73

- Download Model 4
- C-score = -2.68

- Download Model 5
- C-score = -1.99

Figure S1

ASPARTIC (Asp, D)

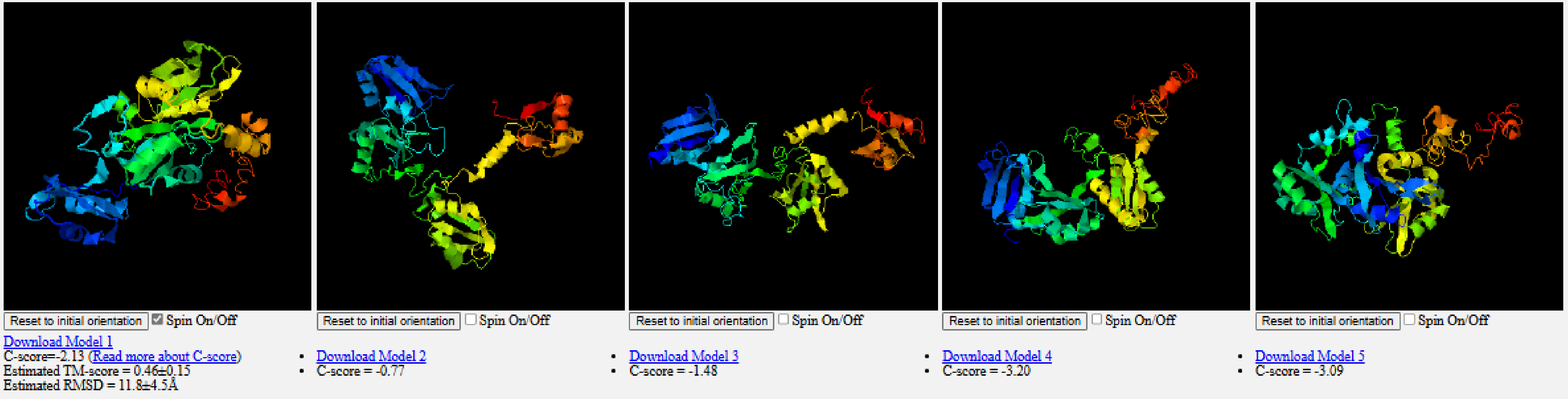

ARGININE (Arg, R)

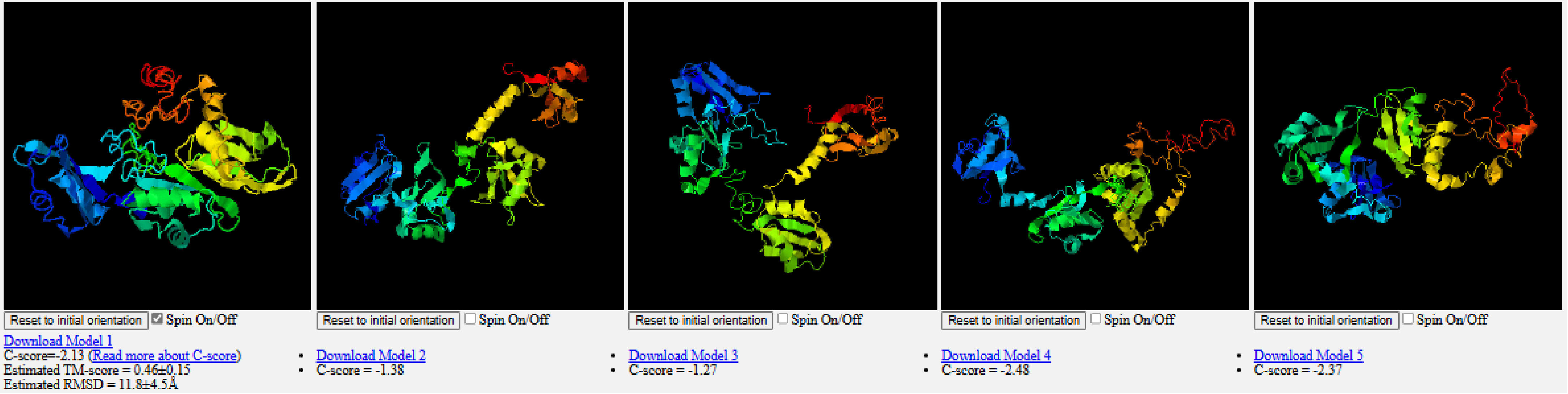

Figure S1

HISTIDINE (His, H)

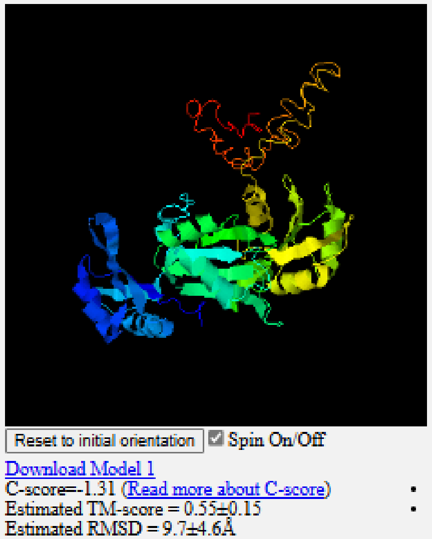

Reset to initial orientation ☑ Spin On/Off

- Download Model 1
- C-score=-1.31 (Read more about C-score)
- Estimated TM-score = 0.55±0.15
- Estimated RMSD = 9.7±4.6Å

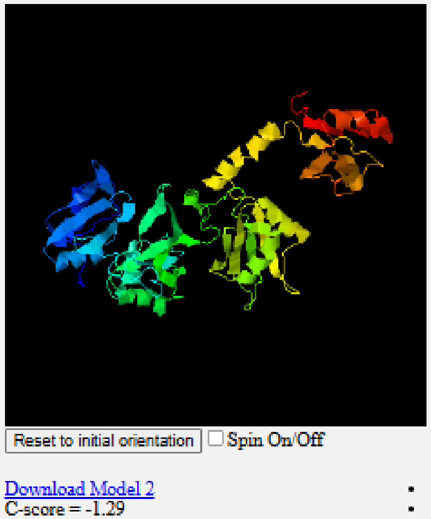

Reset to initial orientation ☐ Spin On/Off

- Download Model 2
- C-score = -1.29

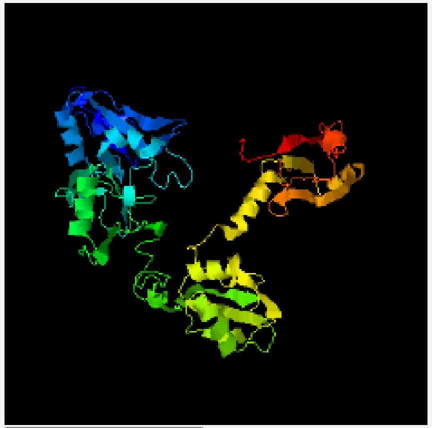

Reset to initial orientation ☐ Spin On/Off

- Download Model 3
- C-score = -2.86

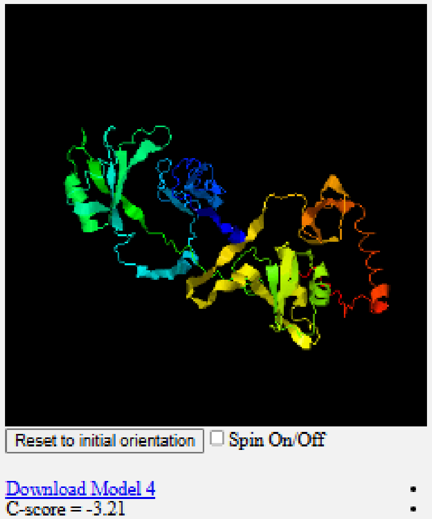

Reset to initial orientation ☐ Spin On/Off

- Download Model 4
- C-score = -3.21

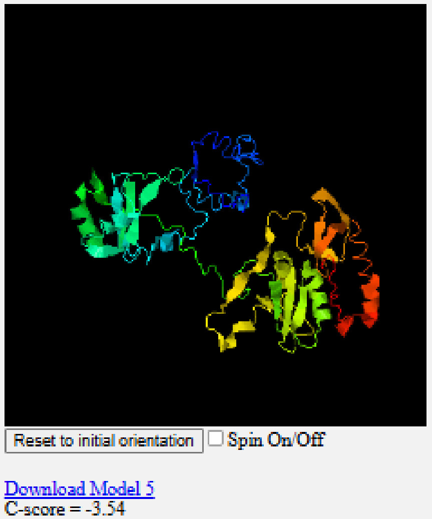

Reset to initial orientation ☐ Spin On/Off

- Download Model 5
- C-score = -3.54

SERINE (Ser, S)

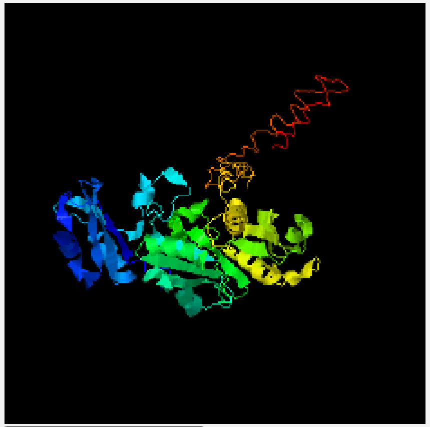

Reset to initial orientation ☑ Spin On/Off

- Download Model 1
- C-score=-2.17 (Read more about C-score)
- Estimated TM-score = 0.46±0.15
- Estimated RMSD = 11.9±4.4Å

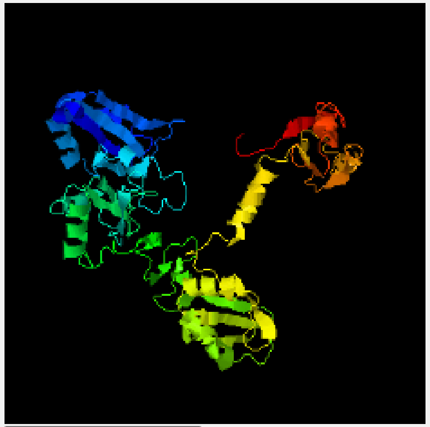

Reset to initial orientation ☐ Spin On/Off

- Download Model 2
- C-score = -0.69

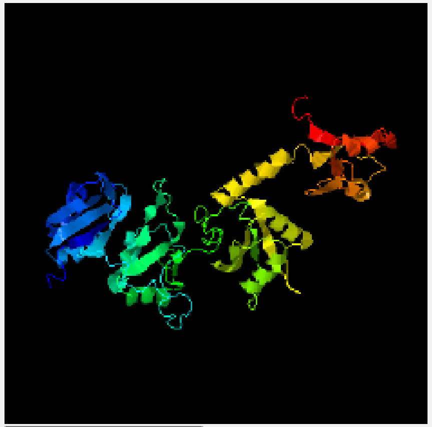

Reset to initial orientation ☐ Spin On/Off

- Download Model 3
- C-score = -0.93

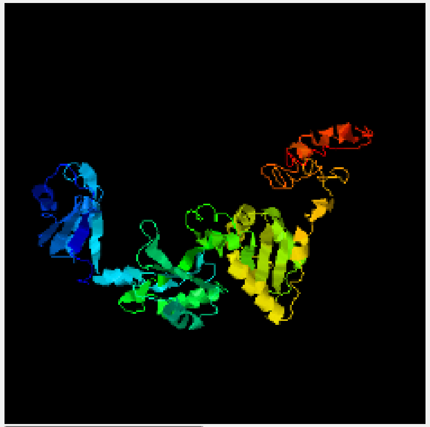

Reset to initial orientation ☐ Spin On/Off

- Download Model 4
- C-score = -2.69

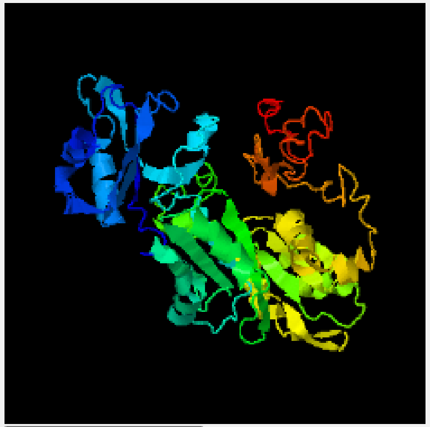

Reset to initial orientation ☐ Spin On/Off

- Download Model 5
- C-score = -3.52

Figure S1

THREONINE (Thr, T)

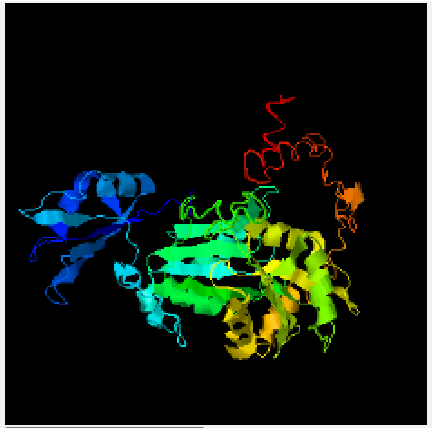

Reset to initial orientation ☑ Spin On/Off

- Download Model 1
- C-score=-0.95 (Read more about C-score)
- Estimated TM-score = 0.59±0.14
- Estimated RMSD = 8.9±4.6Å

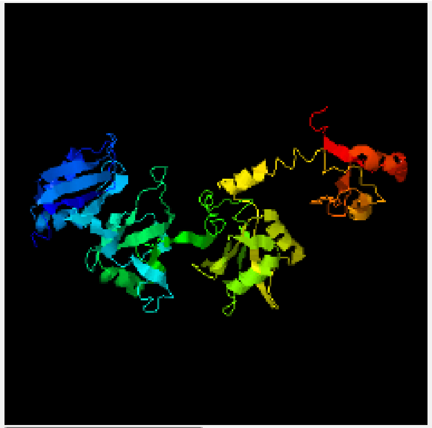

Reset to initial orientation ☐ Spin On/Off

- Download Model 2
- C-score = -1.29

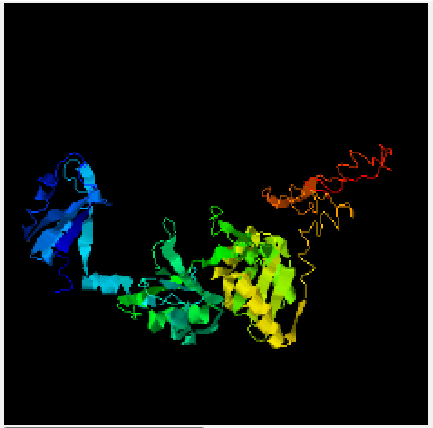

Reset to initial orientation ☐ Spin On/Off

- Download Model 3
- C-score = -2.19

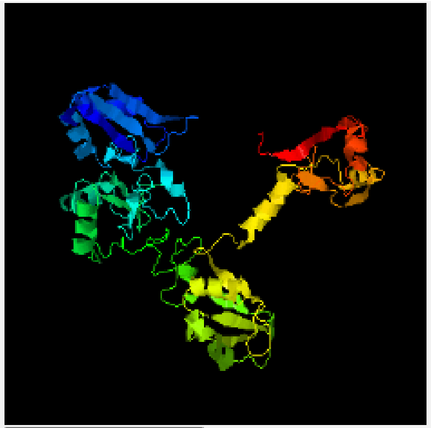

Reset to initial orientation ☐ Spin On/Off

- Download Model 4
- C-score = -3.12

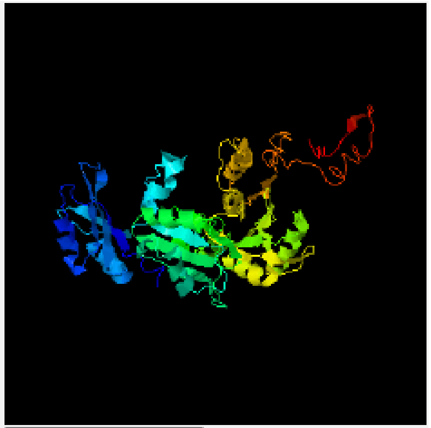

Reset to initial orientation ☐ Spin On/Off

- Download Model 5
- C-score = -3.38

ASPARAGINE (Asn, N)

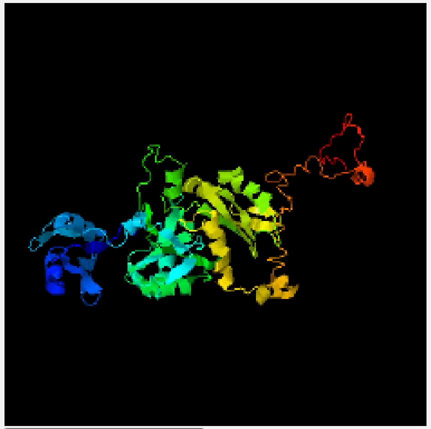

Reset to initial orientation ☑ Spin On/Off

- Download Model 1
- C-score=-2.07 (Read more about C-score)
- Estimated TM-score = 0.47±0.15
- Estimated RMSD = 11.6±4.5Å

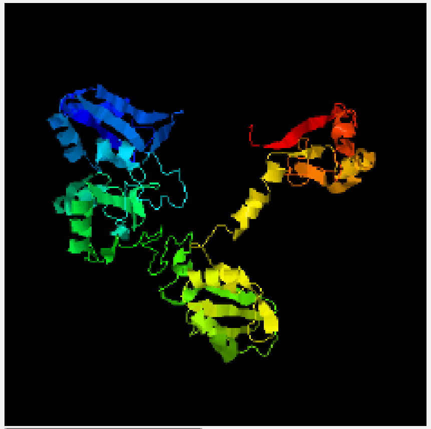

Reset to initial orientation ☐ Spin On/Off

- Download Model 2
- C-score = -0.88

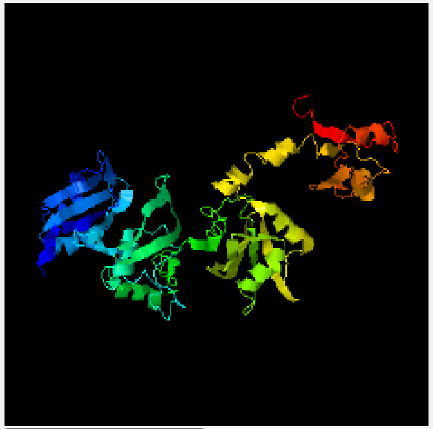

Reset to initial orientation ☐ Spin On/Off

- Download Model 3
- C-score = -0.60

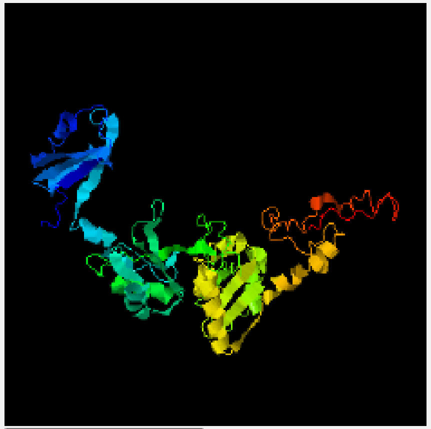

Reset to initial orientation ☐ Spin On/Off

- Download Model 4
- C-score = -3.29

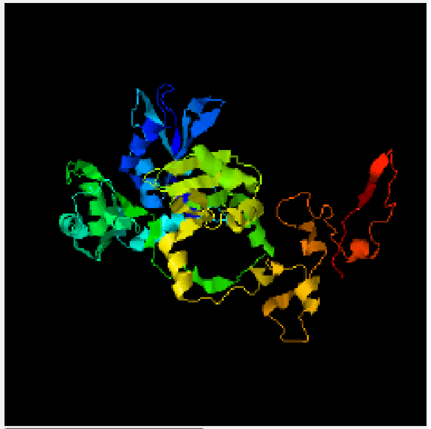

Reset to initial orientation ☐ Spin On/Off

- Download Model 5
- C-score = -2.19

Figure S1

CYSTEINE (Cys, C)

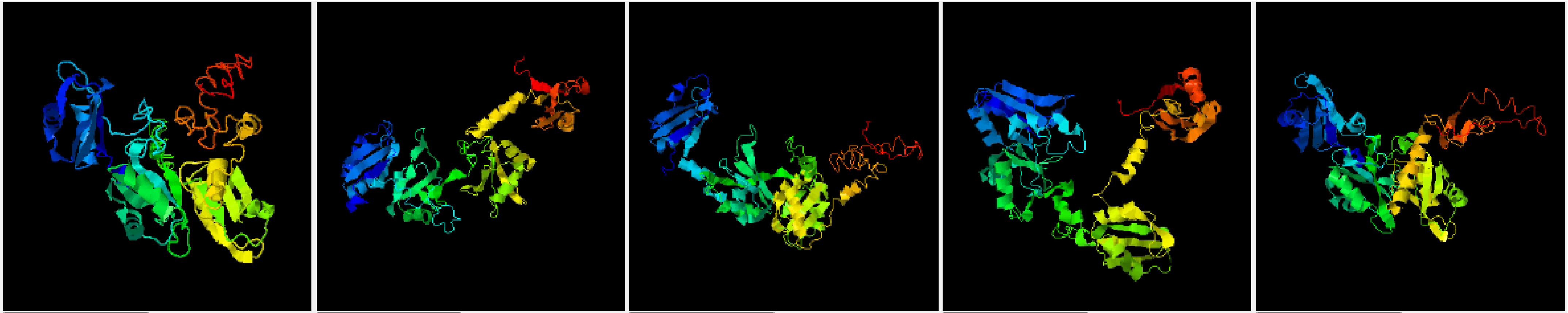

Reset to initial orientation ☑ Spin On/Off

- Download Model 1
- C-score=-1.05 (Read more about C-score)
- Estimated TM-score = 0.58±0.14
- Estimated RMSD = 9.1±4.6Å

Reset to initial orientation ☐ Spin On/Off

- Download Model 2
- C-score = -0.97

Reset to initial orientation ☐ Spin On/Off

- Download Model 3
- C-score = -2.09

Reset to initial orientation ☐ Spin On/Off

- Download Model 4
- C-score = -2.60

Reset to initial orientation ☐ Spin On/Off

- Download Model 5
- C-score = -1.97

GLUTAMINE (Gln, Q)

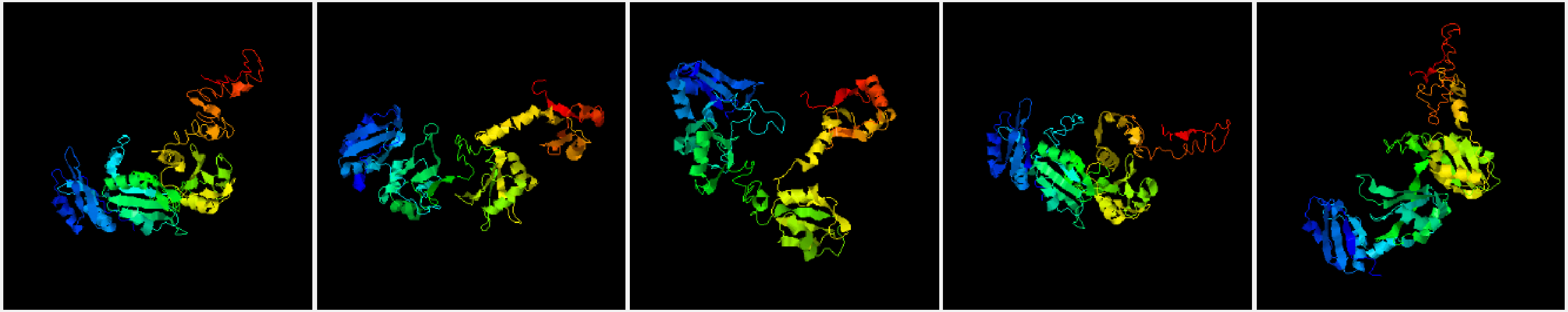

Reset to initial orientation ☑ Spin On/Off

Download Model 1
C-score=-2.04 (Read more about C-score)
Estimated TM-score = 0.47±0.15
Estimated RMSD = 11.6±4.5Å

Reset to initial orientation ☐ Spin On/Off

- Download Model 2
- C-score = -1.45

Reset to initial orientation ☐ Spin On/Off

- Download Model 3
- C-score = -0.99

Reset to initial orientation ☐ Spin On/Off

- Download Model 4
- C-score = -2.80

Reset to initial orientation ☐ Spin On/Off

- Download Model 5
- C-score = -3.06

GLYCINE (Gly, G)

Figure S1

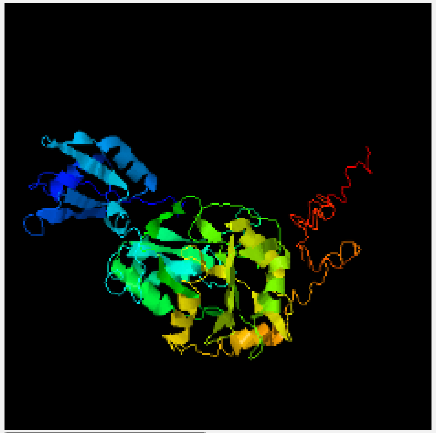

Reset to initial orientation ☑ Spin On/Off

- Download Model 1
- C-score=-2.14 (Read more about C-score)
- Estimated TM-score = 0.46±0.15
- Estimated RMSD = 11.8±4.5Å

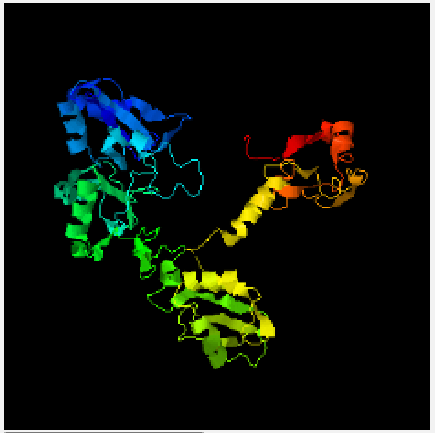

Reset to initial orientation ☐ Spin On/Off

- Download Model 2
- C-score = -0.73

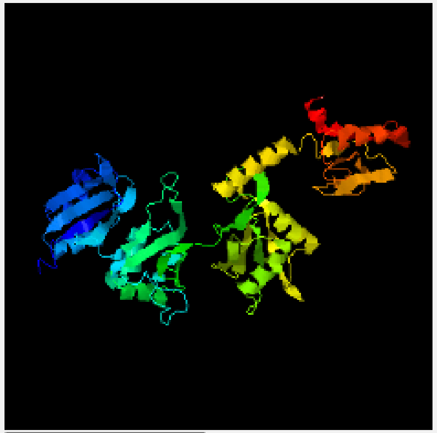

Reset to initial orientation ☐ Spin On/Off

- Download Model 3
- C-score = -0.58

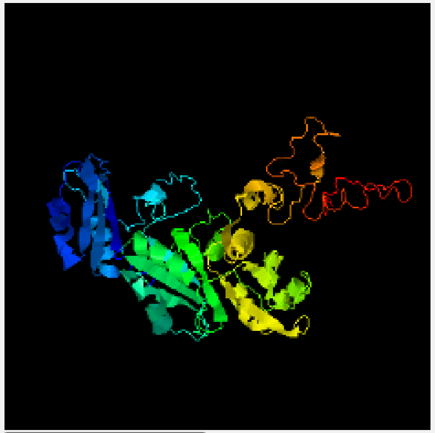

Reset to initial orientation ☐ Spin On/Off

- Download Model 4
- C-score = -3.20

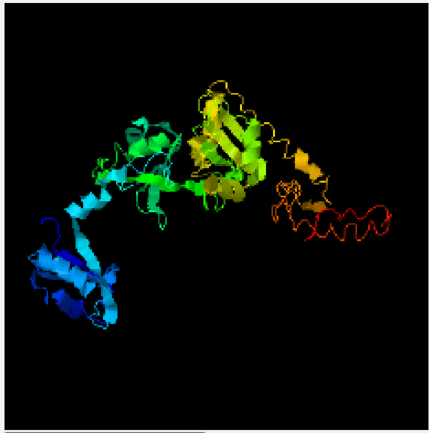

Reset to initial orientation ☐ Spin On/Off

- Download Model 5
- C-score = -3.50

Figure S1

ALANINE (Ala, A)

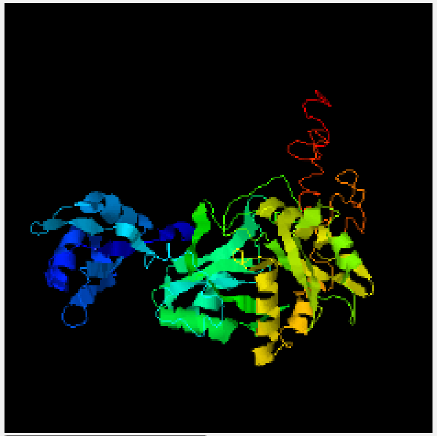

Reset to initial orientation ☑ Spin On/Off

- Download Model 1
- C-score=-2.10 (Read more about C-score)
- Estimated TM-score = 0.46±0.15
- Estimated RMSD = 11.7±4.5Å

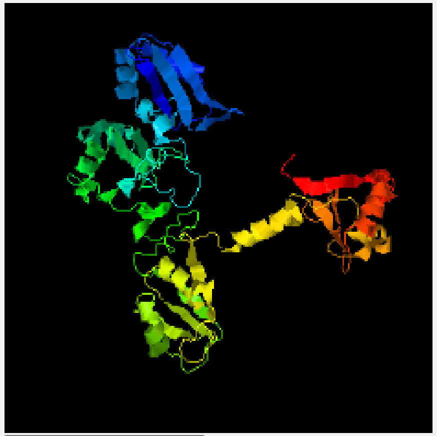

Reset to initial orientation ☐ Spin On/Off

- Download Model 2
- C-score = -0.64

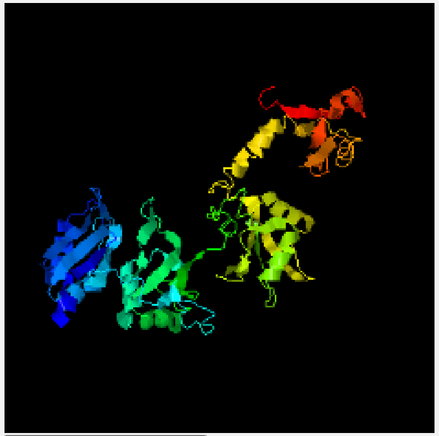

Reset to initial orientation ☐ Spin On/Off

- Download Model 3
- C-score = -0.93

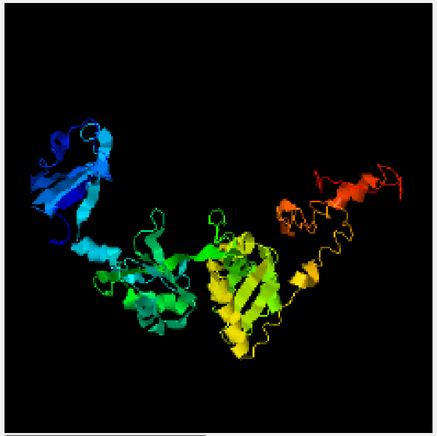

Reset to initial orientation ☐ Spin On/Off

- Download Model 4
- C-score = -3.44

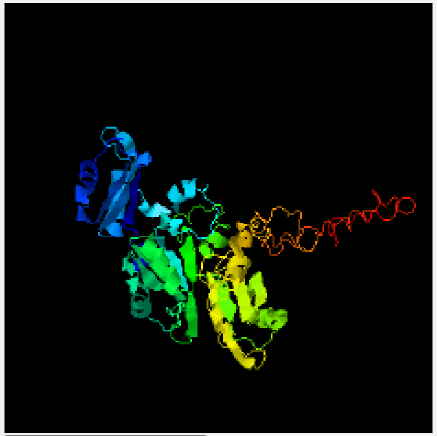

Reset to initial orientation ☐ Spin On/Off

- Download Model 5
- C-score = -3.28

VALINE (Val, V)

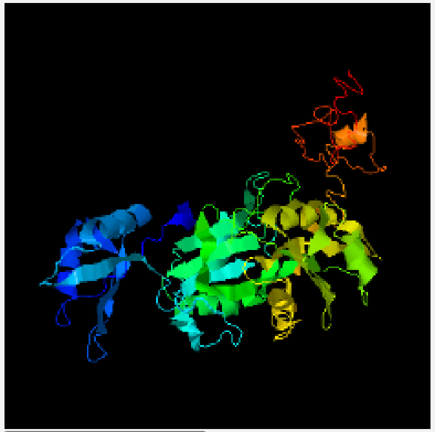

Reset to initial orientation ☑ Spin On/Off

- Download Model 1
- C-score=-0.92 (Read more about C-score)
- Estimated TM-score = 0.60±0.14
- Estimated RMSD = 8.8±4.6Å

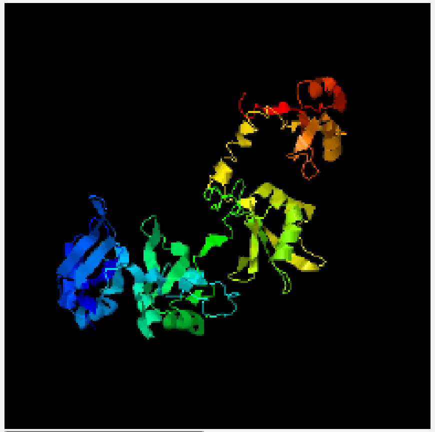

Reset to initial orientation ☐ Spin On/Off

- Download Model 2
- C-score = -0.53

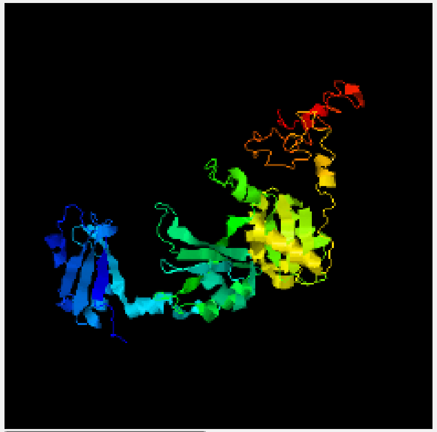

Reset to initial orientation ☐ Spin On/Off

- Download Model 3
- C-score = -2.14

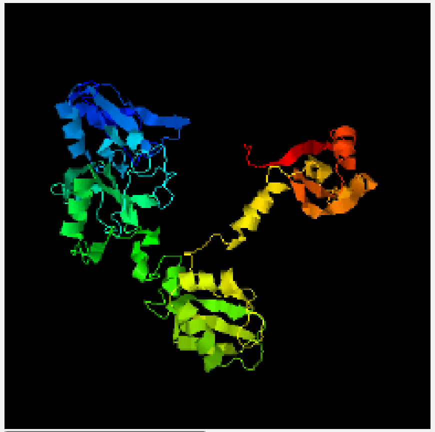

Reset to initial orientation ☐ Spin On/Off

- Download Model 4
- C-score = -3.17

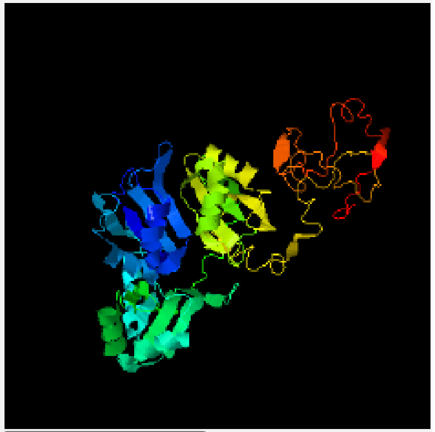

Reset to initial orientation ☐ Spin On/Off

- Download Model 5
- C-score = -2.02

Figure S1

LEUCINE (Leu, L)

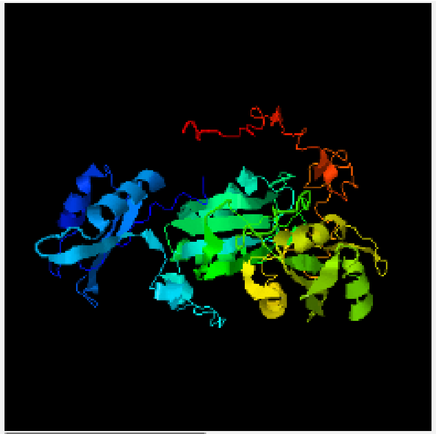

Reset to initial orientation ☑ Spin On/Off

- Download Model 1
- C-score=-0.89 (Read more about C-score)
- Estimated TM-score = 0.60±0.14
- Estimated RMSD = 8.7±4.6Å

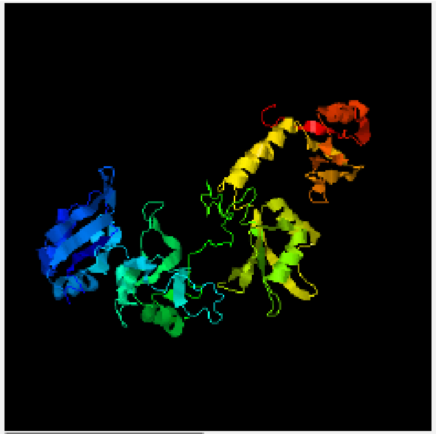

Reset to initial orientation ☐ Spin On/Off

- Download Model 2
- C-score = -1.22

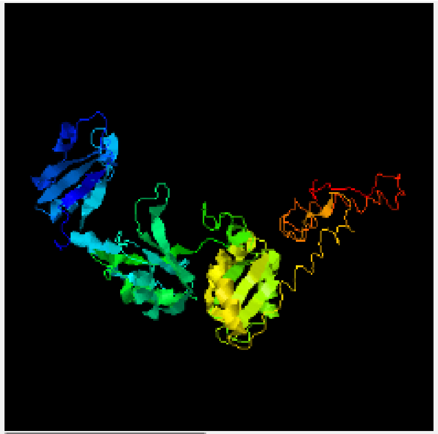

Reset to initial orientation ☐ Spin On/Off

- Download Model 3
- C-score = -2.18

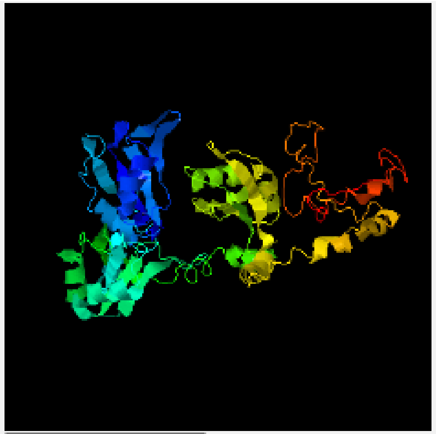

Reset to initial orientation ☐ Spin On/Off

- Download Model 4
- C-score = -2.86

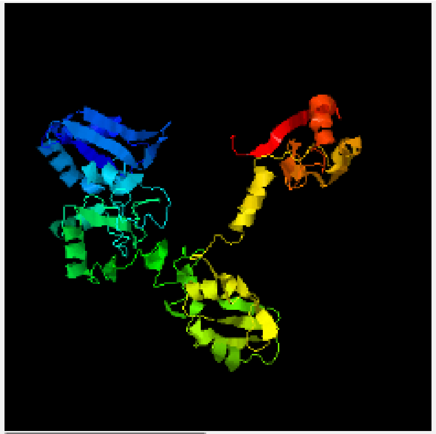

Reset to initial orientation ☐ Spin On/Off

- Download Model 5
- C-score = -3.39

ISOLEUCINE (Ile, I)

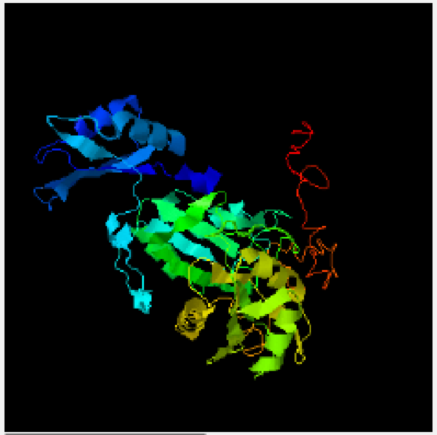

Reset to initial orientation ☑ Spin On/Off

- Download Model 1
- C-score=-1.18 (Read more about C-score)
- Estimated TM-score = 0.57±0.15
- Estimated RMSD = 9.4±4.6Å

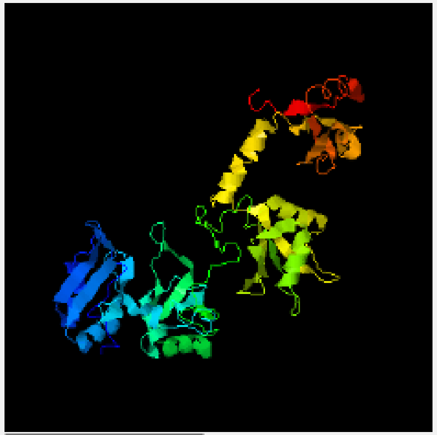

Reset to initial orientation ☐ Spin On/Off

- Download Model 2
- C-score = -1.40

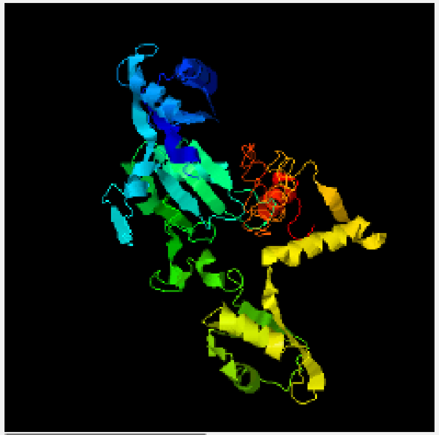

Reset to initial orientation ☐ Spin On/Off

- Download Model 3
- C-score = -1.85

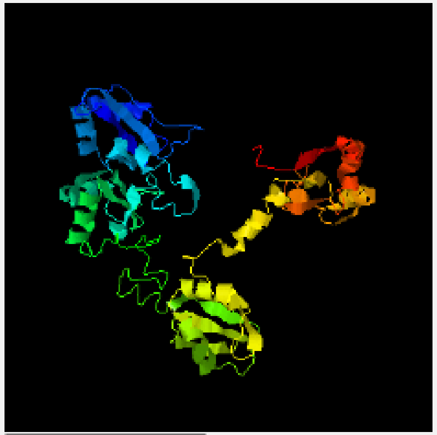

Reset to initial orientation ☐ Spin On/Off

- Download Model 4
- C-score = -3.61

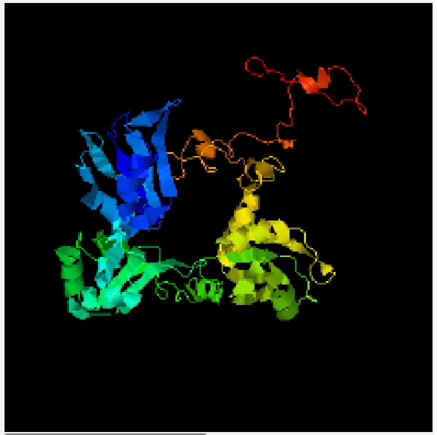

Reset to initial orientation ☐ Spin On/Off

- Download Model 5
- C-score = -2.46

Figure S1

METHIONINE (Met, M)

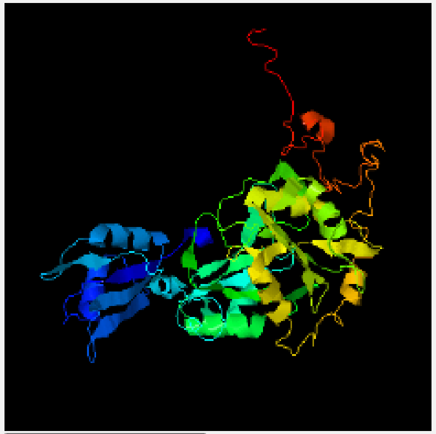

Reset to initial orientation ☑ Spin On/Off

- Download Model 1
- C-score=-0.84 (Read more about C-score)
- Estimated TM-score = 0.61±0.14
- Estimated RMSD = 8.6±4.5Å

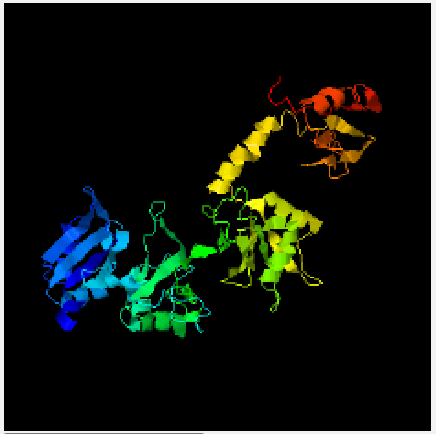

Reset to initial orientation ☐ Spin On/Off

- Download Model 2
- C-score = -0.92

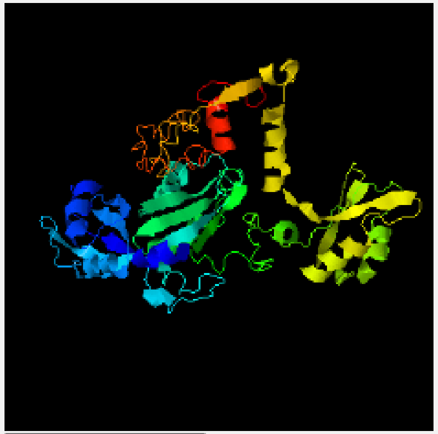

Reset to initial orientation ☐ Spin On/Off

- Download Model 3
- C-score = -2.04

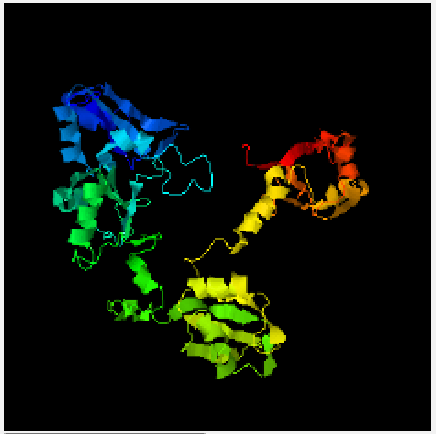

Reset to initial orientation ☐ Spin On/Off

- Download Model 4
- C-score = -3.41

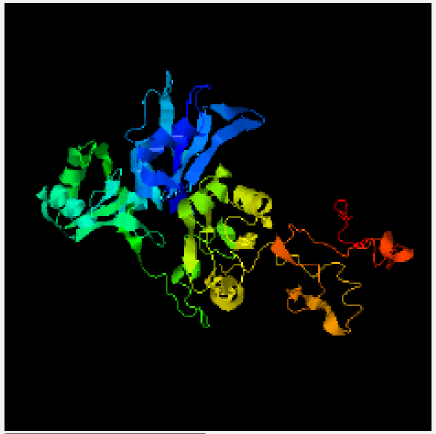

Reset to initial orientation ☐ Spin On/Off

- Download Model 5
- C-score = -2.24

PHENYLALANINE (Phe, F)

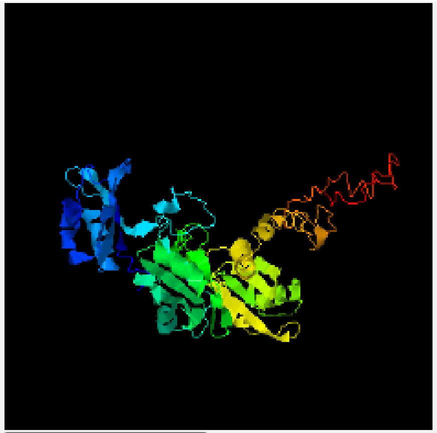

Reset to initial orientation ☑ Spin On/Off

- Download Model 1
- C-score=-1.28 (Read more about C-score)
- Estimated TM-score = 0.56±0.15
- Estimated RMSD = 9.7±4.6Å

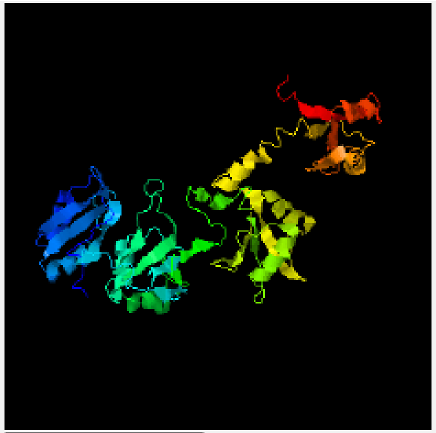

Reset to initial orientation ☐ Spin On/Off

- Download Model 2
- C-score = -1.30

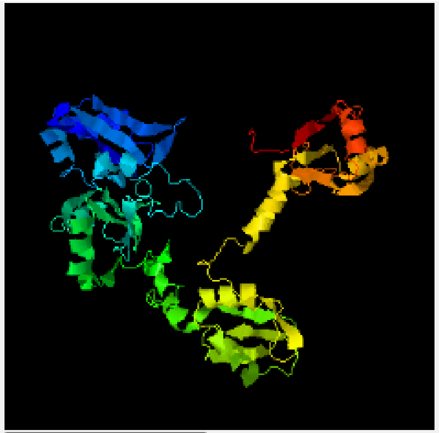

Reset to initial orientation ☐ Spin On/Off

- Download Model 3
- C-score = -2.39

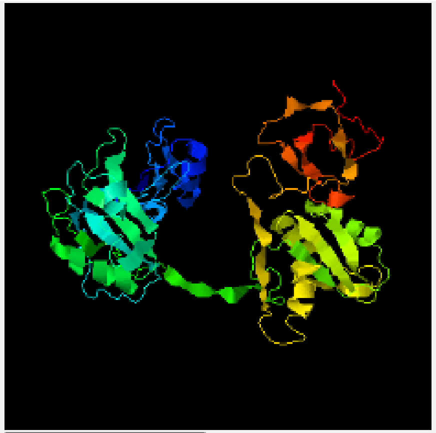

Reset to initial orientation ☐ Spin On/Off

- Download Model 4
- C-score = -3.04

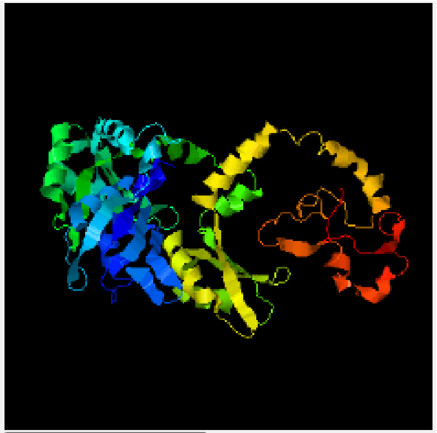

Reset to initial orientation ☐ Spin On/Off

- Download Model 5
- C-score = -2.02

Figure S1

TYROSINE (Tyr, Y)

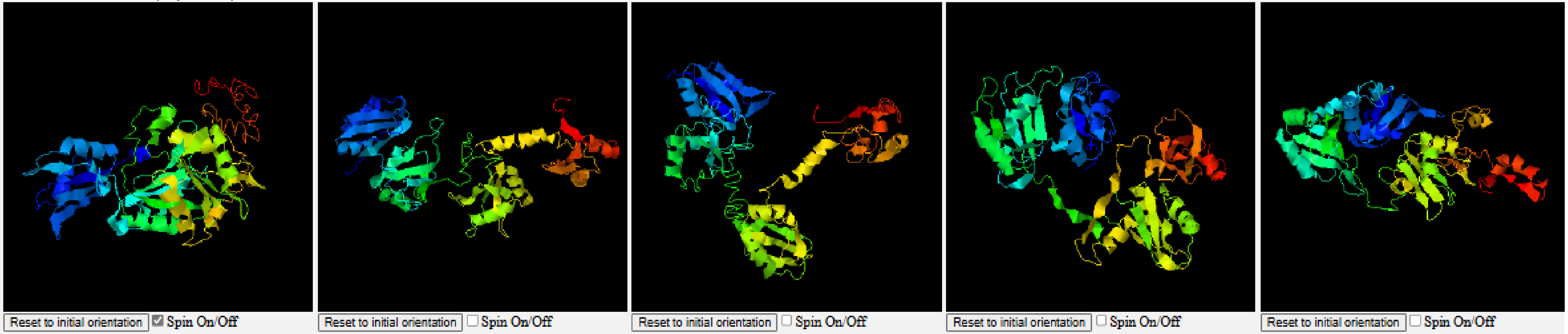

- Download Model 1
- C-score=-1.38 (Read more about C-score)
- Estimated TM-score = 0.54±0.15
- Estimated RMSD = 9.9±4.6Å

- Download Model 2
- C-score = -1.25

- Download Model 3
- C-score = -3.13

- Download Model 4
- C-score = -3.60

- Download Model 5
- C-score = -1.89

TRYPTOPHAN (Trp, W)

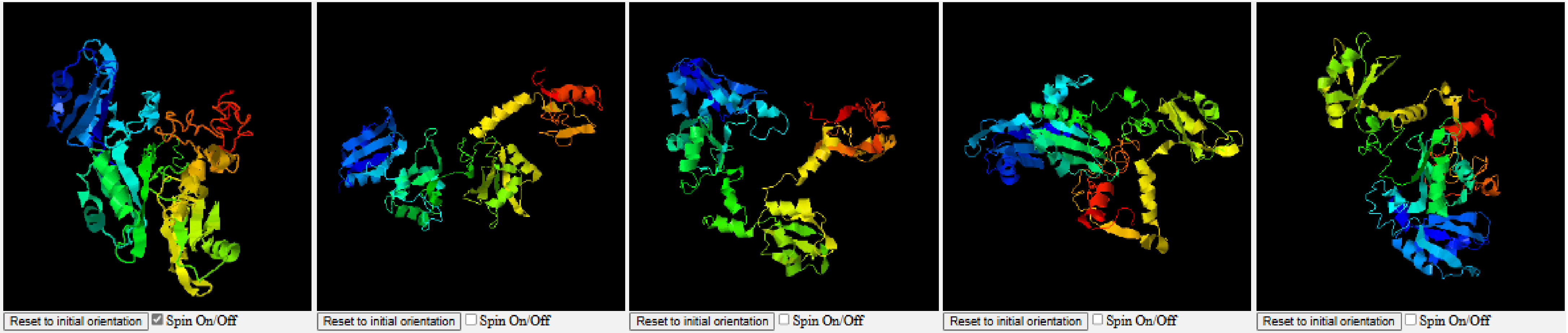

- Download Model 1
- C-score=-0.81 (Read more about C-score)
- Estimated TM-score = 0.61±0.14
- Estimated RMSD = 8.6±4.5Å

- Download Model 2
- C-score = -1.56

- Download Model 3
- C-score = -2.35

- Download Model 4
- C-score = -2.68

- Download Model 5
- C-score = -2.76

Figure S1

PROLINE (Pro, P)

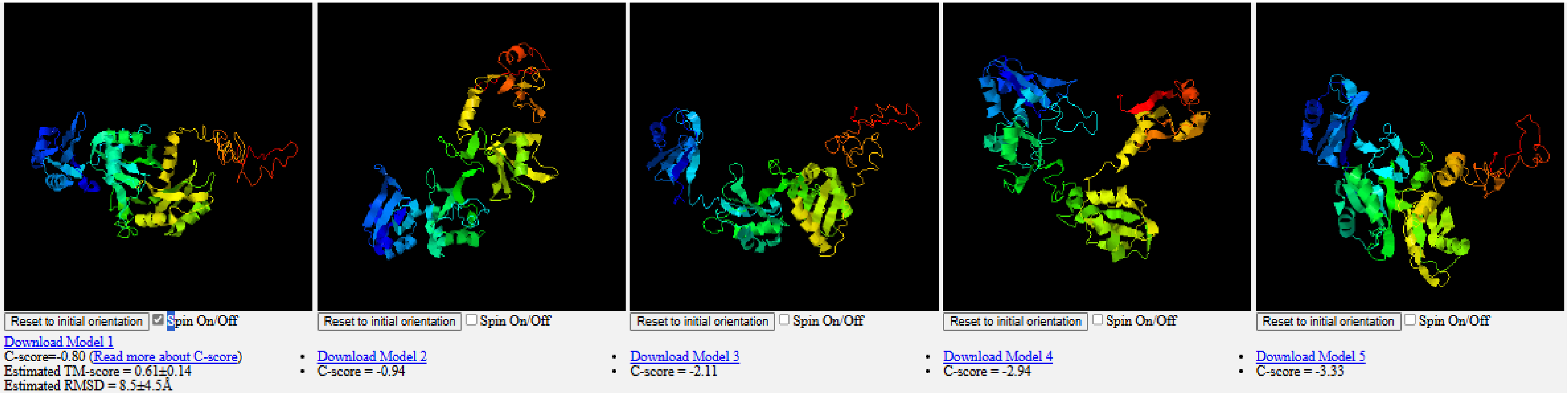

Reset to initial orientation ☑ Spin On/Off

- Download Model 1
- C-score=-0.80 (Read more about C-score)
- Estimated TM-score = 0.61±0.14
- Estimated RMSD = 8.5±4.5Å

Reset to initial orientation ☐ Spin On/Off

- Download Model 2
- C-score = -0.94

Reset to initial orientation ☐ Spin On/Off

- Download Model 3
- C-score = -2.11

Reset to initial orientation ☐ Spin On/Off

- Download Model 4
- C-score = -2.94

Reset to initial orientation ☐ Spin On/Off

- Download Model 5
- C-score = -3.33

Figure S2

GLUTAMINE (Gln, Q)

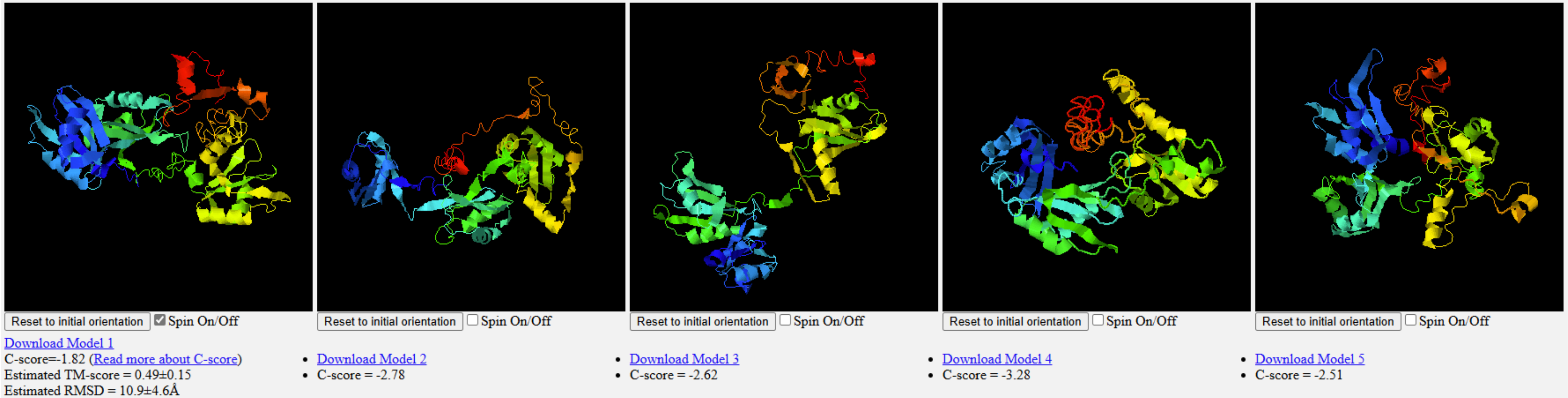

Reset to initial orientation ☑ Spin On/Off

Download Model 1
C-score=-1.82 (Read more about C-score)
Estimated TM-score = 0.49±0.15
Estimated RMSD = 10.9±4.6Å

Reset to initial orientation ☐ Spin On/Off

- Download Model 2
- C-score = -2.78

Reset to initial orientation ☐ Spin On/Off

- Download Model 3
- C-score = -2.62

Reset to initial orientation ☐ Spin On/Off

- Download Model 4
- C-score = -3.28

Reset to initial orientation ☐ Spin On/Off

- Download Model 5
- C-score = -2.51

ASPARTIC ACID (Asp, D)

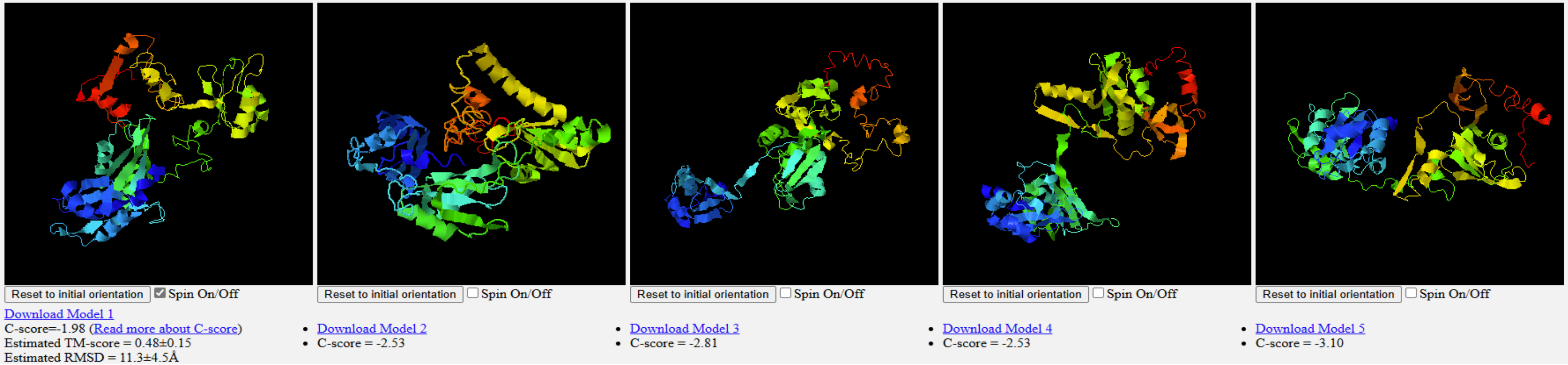

Reset to initial orientation ☑ Spin On/Off

Download Model 1
C-score=-1.98 (Read more about C-score)
Estimated TM-score = 0.48±0.15
Estimated RMSD = 11.3±4.5Å

Reset to initial orientation ☐ Spin On/Off

- Download Model 2
- C-score = -2.53

Reset to initial orientation ☐ Spin On/Off

- Download Model 3
- C-score = -2.81

Reset to initial orientation ☐ Spin On/Off

- Download Model 4
- C-score = -2.53

Reset to initial orientation ☐ Spin On/Off

- Download Model 5
- C-score = -3.10

Figure S2

GLUTAMIC ACID (Glu, E)

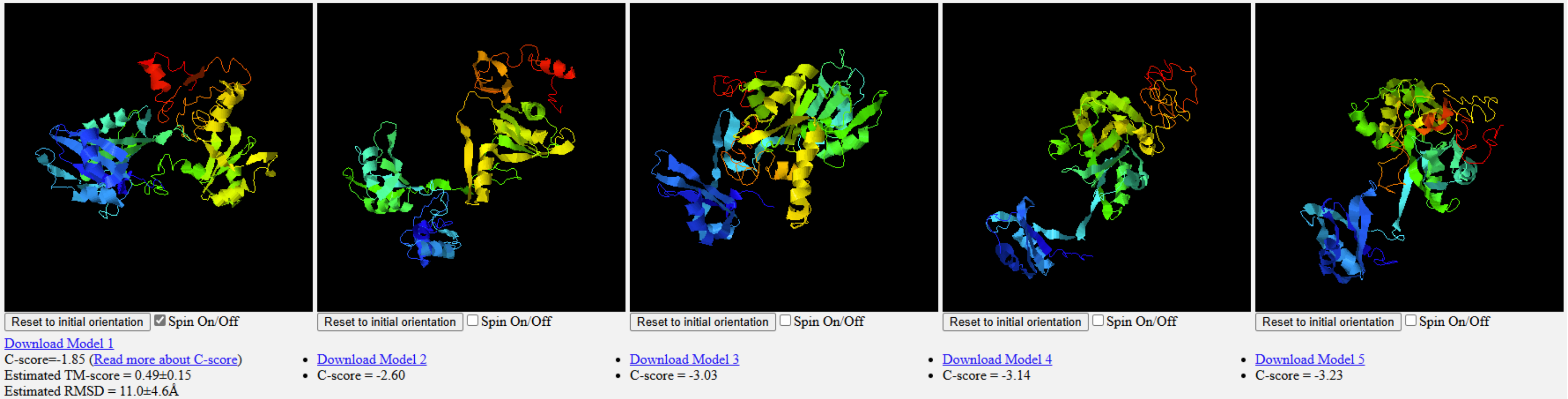

Reset to initial orientation ☑ Spin On/Off | Reset to initial orientation ☐ Spin On/Off | Reset to initial orientation ☐ Spin On/Off | Reset to initial orientation ☐ Spin On/Off | Reset to initial orientation ☐ Spin On/Off

Download Model 1
C-score=-1.85 (Read more about C-score)
Estimated TM-score = 0.49±0.15
Estimated RMSD = 11.0±4.6Å

- Download Model 2
- C-score = -2.60

- Download Model 3
- C-score = -3.03

- Download Model 4
- C-score = -3.14

- Download Model 5
- C-score = -3.23

LYSINE (Lys, K)

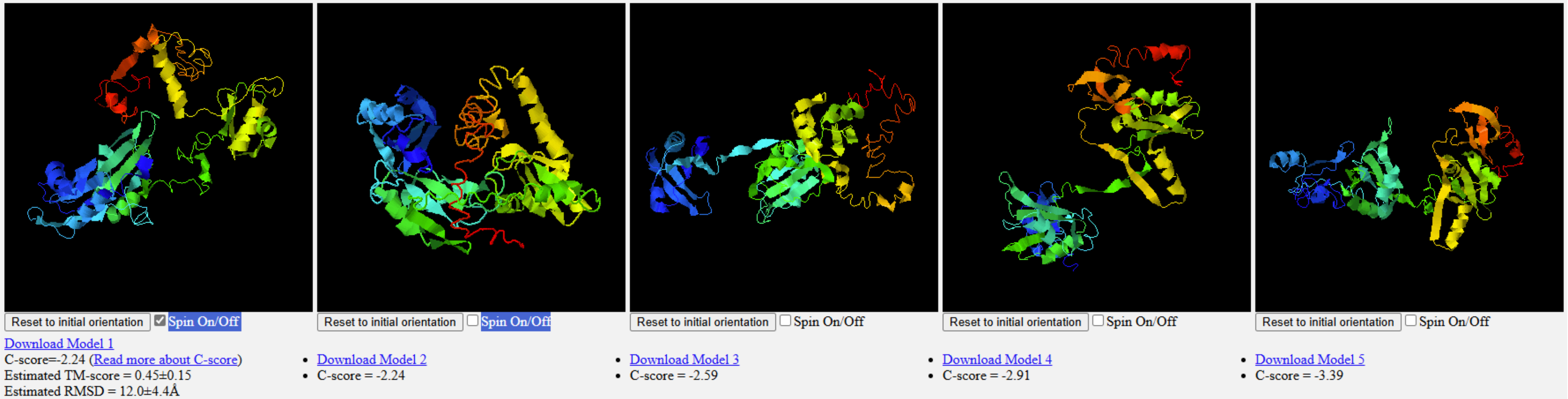

Reset to initial orientation ☑ Spin On/Off | Reset to initial orientation ☐ Spin On/Off | Reset to initial orientation ☐ Spin On/Off | Reset to initial orientation ☐ Spin On/Off | Reset to initial orientation ☐ Spin On/Off

Download Model 1
C-score=-2.24 (Read more about C-score)
Estimated TM-score = 0.45±0.15
Estimated RMSD = 12.0±4.4Å

- Download Model 2
- C-score = -2.24

- Download Model 3
- C-score = -2.59

- Download Model 4
- C-score = -2.91

- Download Model 5
- C-score = -3.39

Figure S2

ARGININE (Arg, R)

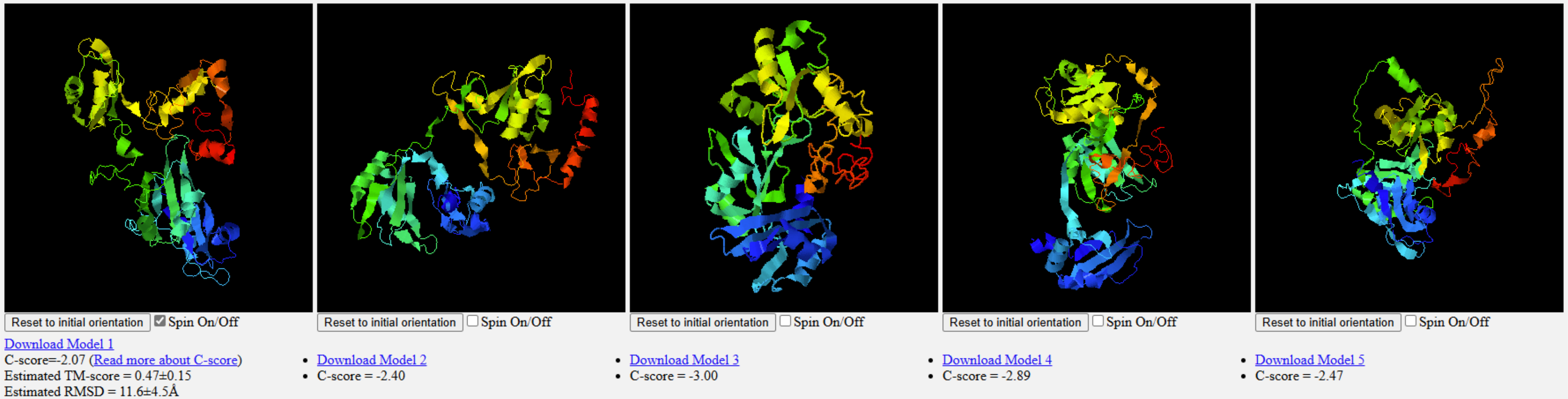

Reset to initial orientation ☑ Spin On/Off | Reset to initial orientation ☐ Spin On/Off | Reset to initial orientation ☐ Spin On/Off | Reset to initial orientation ☐ Spin On/Off | Reset to initial orientation ☐ Spin On/Off

Download Model 1
C-score=-2.07 (Read more about C-score)
Estimated TM-score = 0.47±0.15
Estimated RMSD = 11.6±4.5Å

- Download Model 2
- C-score = -2.40

- Download Model 3
- C-score = -3.00

- Download Model 4
- C-score = -2.89

- Download Model 5
- C-score = -2.47

PROLINE (Pro, P)

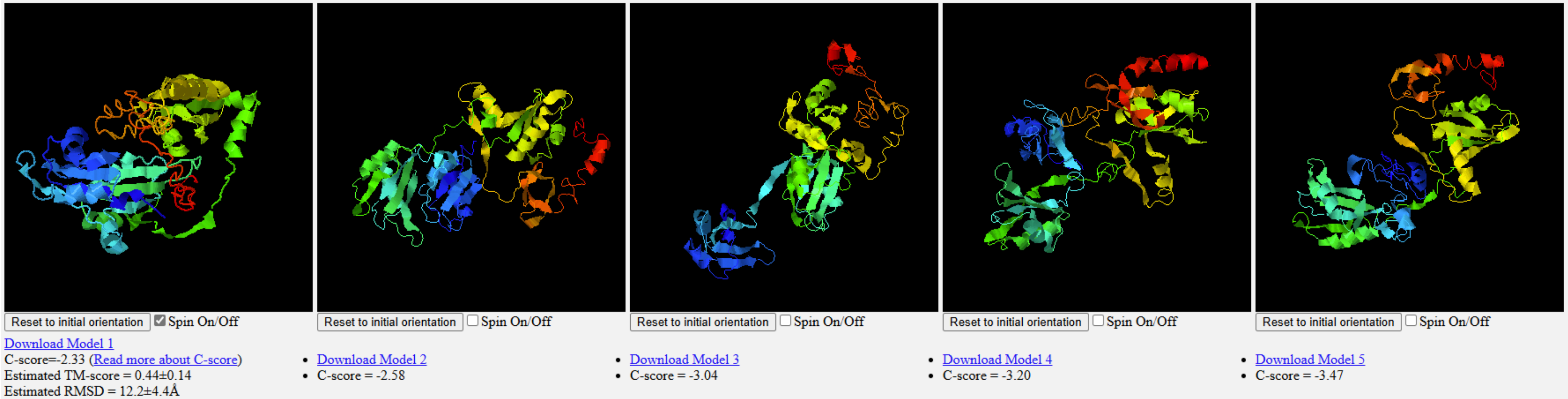

Reset to initial orientation ☑ Spin On/Off | Reset to initial orientation ☐ Spin On/Off | Reset to initial orientation ☐ Spin On/Off | Reset to initial orientation ☐ Spin On/Off | Reset to initial orientation ☐ Spin On/Off

Download Model 1
C-score=-2.33 (Read more about C-score)
Estimated TM-score = 0.44±0.14
Estimated RMSD = 12.2±4.4Å

- Download Model 2
- C-score = -2.58

- Download Model 3
- C-score = -3.04

- Download Model 4
- C-score = -3.20

- Download Model 5
- C-score = -3.47

Figure S3

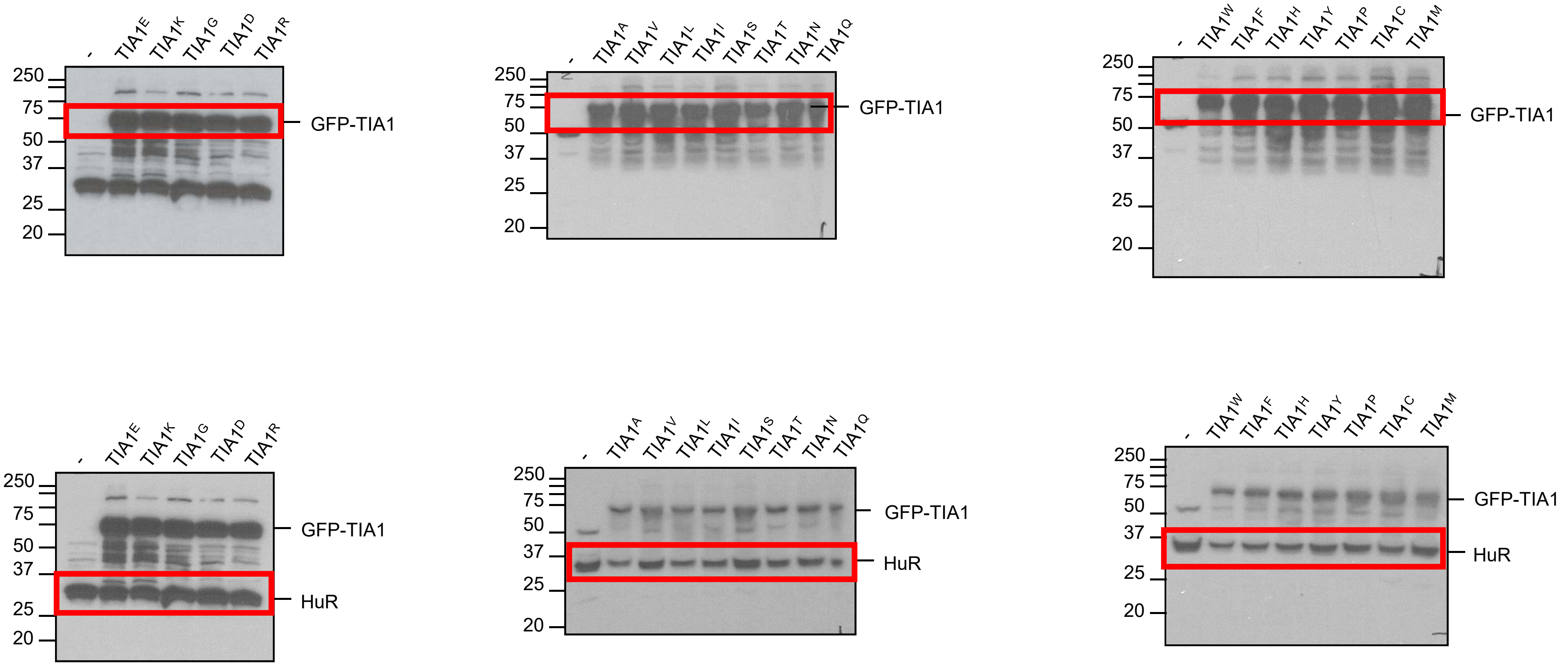

Western blots of Figure 1D

Figure S3

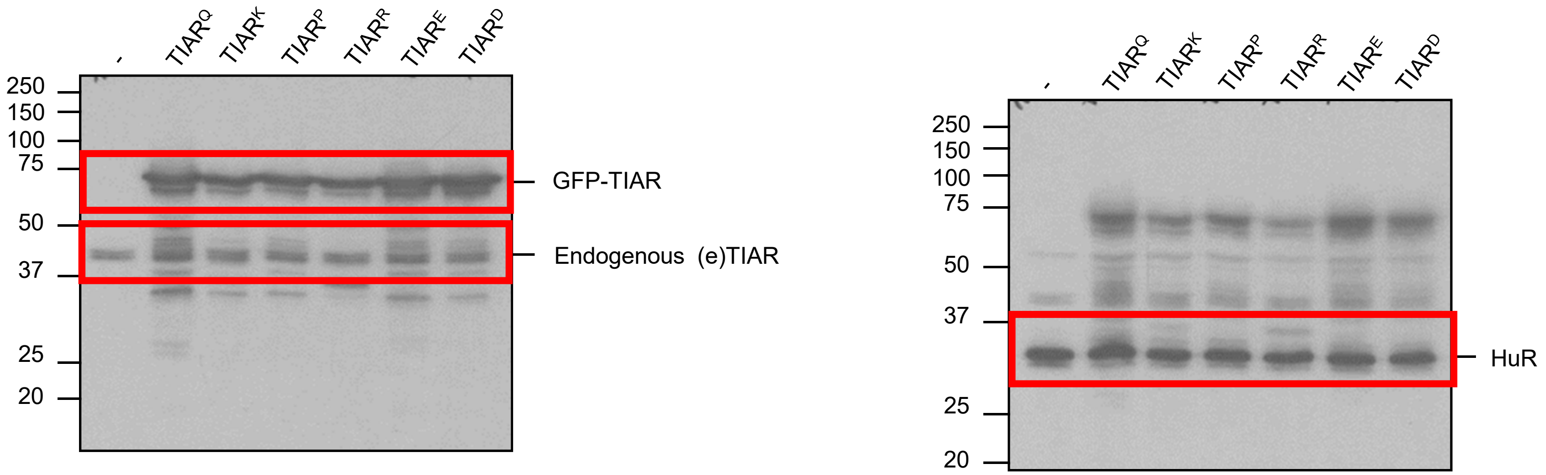

Western blots of Figure 5D

Oligo names	Sequences
TIA1_Asn.sense	5’-AATACCCAGTGAGGATCCACTAGTCCAGTG-3’
TIA1_Gln.sense	5’-CAAACCCAGTGAGGATCCACTAGTCCAGTG-3’
TIA1_Ala.sense	5’-GCAACCCAGTGAGGATCCACTAGTCCAGTG-3’
TIA1_Val.sense	5’-GTAACCCAGTGAGGATCCACTAGTCCAGTG-3’
TIA1_Tyr.sense	5’-TATACCCAGTGAGGATCCACTAGTCCAGTG-3’
TIA1_Trp.sense	5’-TGGACCCAGTGAGGATCCACTAGTCCAGTG-3’
TIA1_His.sense	5’-CATACCCAGTGAGGATCCACTAGTCCAGTG-3’
TIA1_Pro.sense	5’-CCAACCCAGTGAGGATCCACTAGTCCAGTG-3’
TIA1_Met.sense	5’-ATGACCCAGTGAGGATCCACTAGTCCAGTG-3’
TIA1_Ser.sense	5’-TCAACCCAGTGAGGATCCACTAGTCCAGTG-3’
TIA1_Cys.sense	5’-TGTACCCAGTGAGGATCCACTAGTCCAGTG-3’
TIA1_Leu.sense	5’-CTAACCCAGTGAGGATCCACTAGTCCAGTG-3’
TIA1_Ile.sense	5’-ATAACCCAGTGAGGATCCACTAGTCCAGTG-3’
TIA1_Phe.sense	5’-TTTACCCAGTGAGGATCCACTAGTCCAGTG-3’
TIA1_Thr.sense	5’-ACAACCCAGTGAGGATCCACTAGTCCAGTG-3’
TIA1_Universal.antisense	5’-ATACCCTGCCACTCGATACCCAGAAGG-3’
TIA1_Seq.sense	5’-TGGATGGGACCAAATTATG-3’
TIAR_Lys.sense	5’-AAAACACAGTGAGGATCCACTAGTCCAGTG-3’
TIAR_Pro.sense	5’-CCAACACAGTGAGGATCCACTAGTCCAGTG-3’
TIAR_Arg.sense	5’-CGAACACAGTGAGGATCCACTAGTCCAGTG-3’
TIAR_Glu.sense	5’-GAAACACAGTGAGGATCCACTAGTCCAGTG-3’
TIAR_Asp.sense	5’-GATACACAGTGAGGATCCACTAGTCCAGTG-3’
TIAR_Universal.antisense	5’-GTAACTTGCCATACCATAACCGGCTTG-3’
TIAR_Seq.antisense	5’-GGCAACCTAGAAGGCACAG-3’

Table S1. DNA oligos sequence designed for each of the amino acids to be studied in TIA1 and TIAR proteins and sequences of the oligos used for sequencing the mutant plasmids. The first column shows the mutation carried by the oligos. The second, indicates the sequence of these oligos, being underlined the corresponding triplet to the amino acid to be replaced.
